# Paeoniflorin prevents acute graft-versus-host disease while preserving graft-versus-tumor effects

**DOI:** 10.3389/fimmu.2026.1937379

**Published:** 2026-09-08

**Authors:** Xingyu Wan, Nan Li, Yang Gou, Wuchen Yang, Shengwang Wu, Jing Zhang, Shuiqing Liu, Xi Zhang

**Affiliations:** 1 Medical Center of Hematology, Xinqiao Hospital of Army Medical University, Chongqing, China; 2 Chongqing Key Laboratory of Hematology and Microenvironment, Chongqing, China; 3 Jinfeng Laboratory, Chongqing, China

**Keywords:** acute graft-versus-host disease, allogeneic hematopoietic stem cell transplantation, graft-versus-tumor effect, gut microbiota, hematopoietic reconstitution, paeoniflorin

## Abstract

**Background:**

Acute graft-versus-host disease (aGVHD) remains a major complication after allogeneic hematopoietic stem cell transplantation (allo-HSCT). This study investigated the preventive effects of paeoniflorin (PF), a natural monoterpene glycoside, on aGVHD.

**Methods:**

Candidate herb-derived compounds against aGVHD were screened using SymMap, HERB, ADMETlab, and InflamNat, followed by molecular docking. The preventive effects of PF were evaluated in major histocompatibility complex (MHC)-mismatched murine aGVHD models. An entire-process paeoniflorin (EP-PF) regimen, involving donor pretreatment before transplantation and recipient treatment after transplantation, was proposed for enhancing the preventive effect. Survival, body weight, clinical score, hematopoietic reconstitution, donor T cell infiltration, cytokine and chemokine levels, gut injury, apoptosis, gut microbiota, transcriptomic changes, and graft-versus-tumor (GVT) activity were assessed.

**Results:**

PF was identified as a promising multi-target compound for aGVHD prevention. PF reduced clinical severity, body weight loss, and mortality, while improving hematopoietic recovery and increasing hematopoietic stem/progenitor cell (HSPC) numbers. EP-PF regimen showed stronger protection than recipient-only PF administration. Mechanistically, EP-PF reduced intestinal donor cluster of differentiation 4 (CD4) positive and cluster of differentiation 8 (CD8) positive T cell infiltration, partially restored CD4^+^/CD8^+^ T cell balance, and reversed inflammatory transcriptional programs in donor T cells. Moreover, EP-PF balanced serum levels of cytokines and chemokines, preserved gut barrier integrity, reduced intestinal apoptosis, and partially corrected gut microbiota dysbiosis. Importantly, EP-PF preserved donor cell-mediated tumor control in a green fluorescent protein (GFP)-labeled A20 GVT model. PF also directly inhibited the proliferation of hematologic tumor cells and promoted their apoptosis *in vitro* and *in vivo*.

**Conclusions:**

PF prevents experimental aGVHD through coordinated regulation of donor T cell responses, inflammatory mediators, hematopoietic reconstitution, intestinal barrier injury, apoptosis, and gut microbiota, while preserving GVT activity. These findings support PF as a potential prophylactic candidate for allo-HSCT.

## Introduction

Allogeneic hematopoietic stem cell transplantation (allo-HSCT) is regarded as a curable strategy for hematological disorders and malignancies (1–4). However, acute graft-versus-host disease (aGVHD) remains the major cause of morbidity and mortality after transplantation, with typical lesions of the skin, liver, and gastrointestinal tract due to the attack of normal tissues driven by donor T cells (5). The pathogenesis of aGVHD involves 3 core links, including conditioning regimen-induced damage, subsequent donor T cell activation, and effector T cell-mediated target organ injury. During this process, the release of pro-inflammatory cytokines, such as interferon-gamma (IFN-γ), tumor necrosis factor-alpha (TNF-α), and interleukin-6 (IL-6), together with the activation of key signaling pathways such as the Janus kinase-signal transducer and activator of transcription (JAK-STAT) pathway promotes disease development (6, 7).

Inhibiting or minimizing the activity of donor T cells can effectively prevent the development of aGVHD. Cyclosporin A (CsA), a prototypical calcineurin inhibitor (CNI), is one of the most commonly used immunosuppressants for solid organ transplantation (8). While in the 1980s, it was also applied for aGVHD prevention by blocking donor T cell activation in the early clinical development stage of allo-HSCT (9). Subsequently, studies found that the combination of CsA with short-course, low-dose methotrexate (MTX) exhibited significantly better efficacy, and this regimen became the standard strategy for aGVHD prevention (10). However, it exhibits suboptimal efficacy in haploidentical hematopoietic cell transplantation (Haplo-HCT), pronounced renal toxicity, and increased risk of infection. In recent years, post-transplantation cyclophosphamide (PT-Cy) provides effective prevention for aGVHD in Haplo-HCT (11, 12). However, this approach is limited by delayed hematopoietic reconstitution, elevated risk of viral reactivation, and notable incidence of hemorrhagic cystitis. Despite the development of many treatment options, the incidence of aGVHD after allo-HSCT remains 40%~60%. There is still an urgent need to develop novel, safe, and effective drugs for patients. And we also found that more than 5,000 strategies for aGVHD prevention had been registered on ClinicalTrials.gov, again reflecting the unmet clinical need.

Herbs have a long history of therapeutic use for inflammatory diseases, but their clinical application is limited due to the complexity of components and uncertain functional mechanisms (13, 14). With the development of biotechnology, investigating the bioactive ingredients of molecular functions of herbs has gradually become a research hotspot in the field (15, 16). Compared with multi-component herbs, Single-constituent compounds have unambiguous chemical structures, making it convenient for mechanism investigation, standardized production and clinical application (16, 17). For example, artemisinin, paclitaxel and tanshinone have been widely investigated and clinically applied in malaria, cancer and cardiovascular diseases, respectively (18–22). However, few studies concentrated on the effects of herbal compounds on aGVHD. In this study, we have screened 116 herb-derived single compounds targeting aGVHD based on SymMap and HERB databases. Among them, paeoniflorin (PF) exhibited favorable pharmacokinetic property and the best anti-inflammatory scores, implying its potential for aGVHD prevention.

PF, a natural monoterpene glycoside mainly from *Radix Paeoniae Alba* (Bai Shao) and *Radix Paeoniae Rubra* (Chi Shao), had garnered increasing attention in recent years owing to its anti-inflammatory and immunomodulatory effects (23). For example, PF could attenuate the allergic contact dermatitis symptoms by inhibiting nuclear factor kappa-B (NF-κB) pathway activation and IFN-γ secretion of T cells (24). In lupus nephritis, PF could induce the expansion of regulatory T cells (Tregs) and ameliorate the inflammatory response (25). Moreover, PF was demonstrated to repair injured intestinal epithelial cells and ameliorate ulcerative colitis symptoms by suppressing cell division cycle 42/c-Jun N-terminal kinase (CDC42/JNK) signaling (26), stimulate hematopoietic growth factors and blood cells production after irradiation damage (27, 28), and restore microbial dysbiosis in patients with diabetic cardiomyopathy and ulcerative colitis (29, 30). Abovementioned studies suggested that PF has great capacities in regulating immunity, inflammation and regeneration. However, the effects of PF in preventing aGVHD have not been proven experimentally as yet. In this study, we employed the classic aGVHD mouse models, and demonstrated that PF could prevent aGVHD through suppressing donor T cells, modulating cytokine/chemokine balance, mitigating gut damage and restoring gut microbiota. Importantly, we proposed an optimized entire-process paeoniflorin (EP-PF) management regimen to improve the hematopoietic potential of donor hematopoietic stem/progenitor cells (HSPCs), and promote post-transplant hematopoietic reconstitution. Furthermore, PF preserved the graft-versus-tumor (GVT) effect against hematological tumor cells.

## Materials and methods

### Screening of active herbal compounds

Herbal compounds were sourced from SymMap (http://www.symmap.org/) and HERB (http://herb.ac.cn/) databases (31, 32). ADMETlab platform (https://admetlab3.scbdd.com/) was used to assess pharmacokinetic parameters of herbal compounds based on a multi-task directed message passing neural network (DMPNN) model (33). The following molecular descriptors were considered: molecular weight (MW), the logarithm of the octanol-water partition coefficient (logP), the number of hydrogen-bond acceptors (Hacc), the number of hydrogen-bond donors (Hdon), and topological polar surface area (TPSA). Compounds were then screened by Lipinski Rule (MW ≤ 500 Da; logP ≤ 5; Hacc ≤ 10; Hdon ≤ 5; 1 violation was acceptable), Pfizer Rule (logP ≤ 3; TPSA ≥ 75 Å²; 1 violation was acceptable), excretion suitability (plasma clearance ≤ 5 mL/min/kg), and structural alerts (no acute toxicity, genotoxic carcinogenicity, or non-genotoxic carcinogenicity). The anti-inflammation scores predicted by InflamNat platform based on a multilayer perceptron model (34) were used for evaluating anti-inflammatory activities. Correlation network analysis was visualized by Cytoscape software (version 3.10.2). Molecular docking simulations were carried out by AutoDock Vina software (version 1.2.7), and the binding energy < -5 kcal/mol was regarded as a favorable combination.

### Mice

C57BL/6 (B6, 6 weeks, male), BALB/c (6 weeks, male), and BALB/c-nude (6 weeks, female) mice were purchased from Vital River Laboratory Animal Technology Co. Ltd (Beijing, China). B6 and BALB/c mice were used for aGVHD and GVT models, and BALB/c-nude mice were used for subcutaneous tumor xenograft. Mice were maintained under specific pathogen-free (SPF) conditions at the Experimental Animal Center of Army Medical University (Chongqing, China). Mice had free access to standard rodent chow and clean drinking water, and were housed under controlled environmental conditions (temperature: 22 ± 2 °C; humidity: 50~60%; 12 h light/dark cycle). Animal experiments were conducted in strict accordance with the National Institutes of Health Guide for the Care and Use of Laboratory Animals and Animal Research Reporting of *In Vivo* Experiments (ARRIVE) guidelines. At the end of the experiment, mice were euthanized by carbon dioxide (CO_2_) inhalation using a gradual-fill method (30%~70% chamber volume/min). CO_2_ exposure was maintained for 1–2 minutes after respiratory arrest, followed by cervical dislocation to confirm death.

### Drug preparation

PF (#HY-N0293, MedChemExpress) and CsA (#HY-B0579, MedChemExpress) were supplied as powders. PF powder was stored at -20°C, whereas CsA was stored at 4°C and protected from light. For *in vivo* administration, PF was freshly dissolved in sterile 0.9% saline at concentrations of 1.5 mg/mL (for paeoniflorin low-dose, PF-LD), 3.0 mg/mL (for paeoniflorin medium-dose, PF-MD), and 6.0 mg/mL (for paeoniflorin high-dose, PF-HD). CsA was freshly prepared in corn oil (#HY-Y1888, MedChemExpress) at a concentration of 0.5 mg/mL. All *in vivo* drug formulations were prepared immediately before each administration and were not stored after preparation. For *in vitro* experiments, PF was freshly dissolved in sterile water and diluted with complete culture medium to final concentrations of 10, 25, 50, 100, 200, and 400 μM.

### aGVHD mouse model and PF administration

The mouse aGVHD was induced by transplanting major histocompatibility complex (MHC)-mismatched donor cells into recipient mice according to previously described methods (35, 36). Two different mouse models were applied: B6-to-BALB/c (Donor: B6; Recipient: BALB/c) and BALB/c-to-B6 (Donor: BALB/c; Recipient: B6). Recipient mice underwent lethal total-body irradiation with cobalt-60 γ-ray at day 0, administered in two equal fractions separated by 4 h. BALB/c recipients received two 4.0 Gy fractions (total dose: 8.0 Gy), whereas B6 recipients received two 4.5 Gy fractions (total dose: 9.0 Gy). Subsequently, bone marrow cells (BMCs) were harvested from donor mice and injected into recipient mice via the tail vein within 8 hours (BALB/c recipients: 5 × 10^6^ BMCs; B6 recipients: 8 × 10^6^ BMCs). Recipients receiving BMCs alone constituted the bone marrow transplantation (BMT) control group, whereas aGVHD was induced by the co-infusion of donor splenic cells (SPCs) (BALB/c recipients: 5 × 10^6^ SPCs; B6 recipients: 1 × 10^7^ SPCs). T cell proportion in graft cells was evaluated by flow cytometry before transplantation. Bone marrow donor chimerism (B6 donors: H-2Kb^+^; BALB/c donors: H-2Kd^+^) was assessed at the experimental endpoint or upon study termination due to death.

To evaluate the preventive effects of PF, aGVHD mice were assigned into PF-LD (15 mg/kg/day), PF-MD (30 mg/kg/day), PF-HD (60 mg/kg/day), CsA group (5 mg/kg/day), and aGVHD group. Among them, CsA, a widely used clinical immunosuppressant for aGVHD prophylaxis, was employed as positive drug control. According to the experimental set-up, PF and CsA were administered intraperitoneally once daily from day 1 to day 14 after transplantation. An EP-PF group was also included in the B6-to-BALB/c model, in which donor B6 mice were administered PF (30 mg/kg/day) for 1 week before transplantation, followed by continuous administration of recipient mice with PF (30 mg/kg/day) for 2 weeks (days 1~14) after transplantation.

### Evaluation of aGVHD severity

The severity of aGVHD was evaluated based on survival curves, body weight changes, and clinical aGVHD scores. Survival status of recipients was monitored daily, while clinical aGVHD score and body weight were assessed every 2 days. The criteria of clinical aGVHD score evaluation was outlined in **Supplementary Table 1**, referring to body weight loss, posture, activity, fur texture, and skin integrity (37). Assessment of aGVHD severity continued until day 40 after transplantation.

### GVT mouse model

The GVT mouse model was constructed based on previously reported methods with minor modifications (38, 39). BALB/c recipient mice were randomly assigned to the BMT+Tumor, GVT, and GVT+EP-PF groups. Recipients were irradiated with cobalt-60 γ-ray, delivered as two 4.0 Gy fractions separated by 4 h (total dose: 8.0 Gy) at day 0. Within 8 h after the second irradiation fraction, all recipients were intravenously infused with 5 × 10^6^ B6 donor-derived BMCs and 5 × 10^6^ green fluorescent protein (GFP)-labeled A20 cells. To induce the GVT effects, mice in the GVT and GVT+EP-PF groups were additionally infused with 2 × 10^6^ SPCs. The preparation, storage, working concentration, vehicle, and administration schedule of PF in the GVT+EP-PF group were identical to those described above for the EP-PF group in the aGVHD model. Survival status was monitored daily until day 20 after transplantation. GFP^+^ cell proportions in peripheral blood were assessed at day 5, day 10, and day 15 using flow cytometry.

### Blood cell counting

Peripheral blood was collected from the lateral tail vein of recipient mice at day 0, day 7, day 14, and day 21 after transplantation. Blood cell counts were analyzed using an automated hematology analyzer (DxH 900, Beckman Coulter, USA).

### Liver and renal function tests

Peripheral blood was obtained from mice at 14 days after transplantation, and centrifuged (4°C, 3,000 ×g, 20 min) to separate supernatant serum. Liver and renal function were analyzed using a fully automated biochemistry analyzer (AU5800, Beckman Coulter, USA).

### Pre-administration assay in donor mice

B6 mice were intraperitoneally injected with normal saline or PF (30 mg/kg/day) prepared as described above for 7 days. The number of bone marrow HSPCs, phenotypically defined as lineage (Lin) negative, stem cell antigen-1 (Sca-1) positive, and KIT proto-oncogene receptor tyrosine kinase (c-Kit) positive, collectively termed Lin^-^Sca-1^+^c-Kit^+^ (LSK) cells, was determined by flow cytometry. The messenger RNA (mRNA) expression of *Runx1*, *Tal1*, and *Erg* were determined by real-time quantitative polymerase chain reaction (qPCR). HSPCs were sorted by flow cytometry and used for transcriptome sequencing analysis.

### Cytokine detection

At day 14 after transplantation, mice were placed under deep isoflurane anesthesia for terminal procedure, confirmed by the absence of pedal withdrawal reflex. Approximately 600 μL of whole blood was then collected by cardiac puncture, after which the mice were euthanized by gradual-fill CO_2_ inhalation and death was confirmed by cervical dislocation. Blood was allowed to clot at room temperature for 30 min and centrifuged at 3,000 ×g for 20 min at 4 °C to separate supernatant serum. Enzyme-linked immunosorbent assay kits were used to measure the levels of serum cytokines, including TNF-α (#EK282HS, Multi Sciences), IFN-γ (#EK280HS, Multi Sciences), interleukin-2 (IL-2, #EK202HS, Multi Sciences), IL-6 (#EK206HS, Multi Sciences), interleukin-17A (IL-17A, #EK217HS, Multi Sciences), interleukin-4 (IL-4, #EK204HS, Multi Sciences), interleukin-10 (IL-10, #PI523, Beyotime), C-C motif chemokine ligand 2 (CCL2, #EK287HS, Multi Sciences), C-C motif chemokine ligand 3 (CCL3, #MMA00, R&D Systems), C-C motif chemokine ligand 4 (CCL4, #EK262, Multi Sciences), C-C motif chemokine ligand 5 (CCL5, #EK2129, Multi Sciences), and C-X-C motif chemokine ligand 1 (CXCL1, #MKC00B, R&D Systems), according to manufacturer’s instructions. Absorbance was measured using a SpectraMax i3x Multi-Mode Microplate Reader (Molecular Devices, USA).

### Hematoxylin and eosin staining

Tissues (skin, liver, small intestine, colon, and lung) were collected at 14 days after transplantation, and then fixed in 4% paraformaldehyde (PFA), embedded in paraffin, and sectioned with a microtome (RM2235, Leica, Japan) at 4 μm. Subsequently, sections were deparaffinized, and stained with hematoxylin and eosin (H&E) according to standard protocols. Histological images were captured using a light microscope (BX63, Olympus, Japan). Pathological scores were evaluated according to previously described criteria with minor modifications (**Supplementary Table 2**) (38, 40).

### Real-time quantitative PCR

Total RNA was extracted with RNAiso Plus (#9109, Takara, Japan), and quantified using a spectrophotometer (Nanodrop 2000, ThermoFisher, USA). Reverse transcription was performed with PrimeScript™ RT Reagent Kit (#RR047A, Takara, Japan). Real-time qPCR with SYBR Green qPCR Master Mix (#HY-K0523, MedChemExpress) was carried out on a qPCR instrument (CFX connect, Bio-Rad, USA). The primer sequences were listed in **Supplementary Table 3**.

### Flow cytometry

For the preparation of single cell suspensions used for flow cytometric analysis, bone marrow was flushed from the bones with phosphate-buffered saline (PBS), gently mashed, filtered through a 70 μm cell strainer, and treated with red blood cell lysis buffer. Moreover, the gut tissues were opened longitudinally, washed with cold PBS, cut into small pieces, and incubated in Hanks’ balanced salt solution (HBSS) containing ethylenediaminetetraacetic acid (EDTA) and dithiothreitol to remove epithelial cells. The remaining tissues were digested with collagenase IV and deoxyribonuclease I (DNase I) at 37 °C, filtered using a 70 μm strainer, and enriched by 40%/80% Percoll reagent. Cells were collected in flow cytometry tubes and centrifuged at 1,500 rpm for 3 min to remove supernatants, washed with staining buffer (#C1711, Beyotime), and stained with specific panel of flow cytometry antibodies at 4°C in the dark, following the manufacturer’s recommended protocol. Data acquisition was conducted using a BD LSR Fortessa X-20 flow cytometer (BD Biosciences, USA), and analyzed with FlowJo software (v 10.8.1). For bone marrow chimerism analysis, cells were stained with H-2Kb (#12-5958-82, eBioscience) or H-2Kd (#12-5998-83, eBioscience) antibodies. For LSK cell detection, cells were stained with the antibody combination of hematopoietic lineage antibody cocktail (#22-7770-72, eBioscience), Sca-1 (#17-5981-83, eBioscience), and c-Kit (#12-1171-83, eBioscience). For the detection of donor-derived cluster of differentiation 4 (CD4) positive T cells, cluster of differentiation 8 (CD8) positive T cells, and Tregs, cells were stained with the antibody combination of H-2Kb (#12-5958-82, eBioscience), cluster of differentiation 3 (CD3, #47-0031-82, eBioscience), CD4 (#48-0041-82, eBioscience), CD8 (#46-0081-82, eBioscience), cluster of differentiation 25 (CD25, #564424, BD Pharmingen), and forkhead box P3 (Foxp3, #560401, BD Pharmingen). For the detection of myeloid-derived suppressor cells (MDSCs), cells were stained with the cluster of differentiation 11b (CD11b, #557396, BD Pharmingen) and granulocyte differentiation antigen-1 (Gr-1, #552093, BD Pharmingen) antibodies. *In vitro* experiments, after exposure to 400 μM PF or vehicle for 72 h, cell apoptosis was evaluated using Annexin V-PE (#C1065L, Beyotime) and 7-aminoactinomycin D (7-AAD) (#420404, BioLegend) according to manufacturer’s instructions.

### Transcriptome sequencing

Splenic donor-derived CD4^+^ and CD8^+^ T cells and bone marrow HSPCs were isolated by flow sorting (MoFlo XDP, Beckman Coulter, USA). Total RNA was extracted using RNAiso Plus (#9109, Takara, Japan), and RNA quality was confirmed by an RNA integrity number (RIN) > 8.0 and an A260/A280 absorbance ratio > 1.8. For CD4^+^ and CD8^+^ T cells, complementary DNA (cDNA) libraries were prepared by Ion Total RNA Sequencing Kit v2 (#4479789, Life Technologies), amplified using Ion PI™ Template OT2–200 Kit v2.0 (#4485146, Life Technologies), and sequenced on Ion Proton sequencer (Life Technologies, USA). For HSPCs, cDNA libraries were prepared by NEBNext UltraExpress™ RNA Library Prep Kit (#E3330L, New England Biolabs), amplified using TruSeq PE Cluster Kit v3-cBot-HS (#PE-401-3001, Illumina) for clustering, and sequenced on HiSeq 2500 sequencer (Illumina, USA). Fold change (FC) values were expressed on the log_2_ scale, and differentially expressed genes (DEGs) were identified using DESeq2 with thresholds of |log_2_FC| > 1.0 and adjusted p < 0.05. Gene Ontology (GO) and Kyoto Encyclopedia of Genes and Genomes (KEGG) enrichment analyses were performed using the Database for Annotation, Visualization and Integrated Discovery (DAVID). Gene set enrichment analysis (GSEA) was performed by SRplot platform (41).

### Diarrhea assessment

Diarrhea severity was assessed by diarrhea rate, diarrhea index and fecal moisture at 14 days after transplantation. The indicators were defined as follows (42, 43).

Diarrhea rate = Number of loose stools/Total bowel movements.

Diarrhea index = Diarrhea rate × Diarrhea grade.

Fecal moisture = [1 - (Dry fecal weight/Fresh fecal weight)] × 100%.

### Gut permeability assessment

Gut permeability was assessed at 14 days after transplantation. Mice were fasted for 12 hours, and orally administered with fluorescein isothiocyanate-dextran (FITC-Dextran) at 400 mg/kg (#Y240442, Beyotime). Peripheral blood was collected 4 hours later, and centrifuged (4°C, 3000×g, 20 min) to separate supernatant serum. Fluorescence intensity was detected at 520 nm using a microplate reader (SpectraMax i3x, Molecular Devices, USA). Serum FITC-Dextran concentration was determined based on a standard curve of serially diluted FITC-Dextran. Higher serum FITC-Dextran concentration indicated elevated gut permeability.

### Western blotting assay

Tissue samples were lysed in radioimmunoprecipitation assay (RIPA) lysis buffer (#P0013C, Beyotime) supplemented with 1% protease inhibitor cocktail (#P1005, Beyotime) on ice, centrifuged at 12,000 ×g for 15 min at 4 °C, and the supernatants were collected. Protein concentrations were determined using a bicinchoninic acid (BCA) Protein Assay Kit (#P0010, Beyotime) according to the manufacturer’s instructions. Equal amounts of protein were separated by sodium dodecyl sulfate-polyacrylamide gel electrophoresis (SDS-PAGE) and transferred onto polyvinylidene difluoride (PVDF) membranes (#FFP19, Beyotime). After blocking with 5% non-fat milk for 1 h at room temperature, the membranes were incubated overnight at 4 °C with primary antibodies against Claudin-1 (1:1000, #ab307692, Abcam), Occludin (1:1000, #ab216327, Abcam), Zonula occludens-1 (ZO-1, 1:1000, #ab96587, Abcam), Cleaved Caspase 3 (1:1000, #9661, Cell Signaling Technology), Cleaved Caspase 9 (1:1000, #9509, Cell Signaling Technology), Bax (1:1000, #2772, Cell Signaling Technology), Bcl-2 (1:1000, #ab194583, Abcam), and β-actin (1:1000, #4967, Cell Signaling Technology). The membranes were then washed with Tris-buffered saline with Tween-20 (TBST) (#T1085, Solarbio) and incubated with horseradish peroxidase (HRP)-conjugated secondary antibodies (1:2000, #7074, Cell Signaling Technology) for 1 h at room temperature. Protein bands were visualized using an enhanced chemiluminescence (ECL) detection reagent (#P0018S, Beyotime), and imaged with a chemiluminescence imaging system.

### Gut microbiota 16S ribosomal RNA sequencing

Feces were harvested from mice at 14 days after transplantation. Genomic DNA was extracted using cetyltrimethylammonium bromide (CTAB) (#ST1186, Beyotime), and the V3-V4 hypervariable regions were amplified. Quality and concentration were examined by agarose gel electrophoresis and spectrophotometer analysis. Libraries were constructed and sequenced on NovaSeq PE250 platform (Illumina, USA). Sequencing data were analyzed using Quantitative Insights Into Microbial Ecology 2 (QIIME 2) and python 3.8. Gut microbiota features were described by diversity, linear discriminant analysis effect size (LEfSe) analysis and differential metabolic pathway analysis.

### Cell culture

The HL-60 (#CCL-240), K-562 (#CCL-243), KG-1a (#CCL-246.1) and SU-DHL-6 (#CRL-2959) cell lines were obtained from American Type Culture Collection (ATCC). The GFP-labeled A20 cell line (#CSC-RR0532) was obtained from Creative Biogene (USA). All cells were cultured in their respective recommended media at 37 °C in a humidified incubator with 5% CO_2_.

### Cell counting assay

Cell proliferation was evaluated using Enhanced Cell Counting Kit-8 (CCK-8 kit, #C0043, Beyotime). Cells were seeded in 96-well plates at a density of 20,000 cells/well and incubated for designed time (0, 24, 48 and 72 hours). Then, 10 μL of CCK-8 reagent was added into each well and incubated for 2 h. The absorbance was detected at 450 nm using a microplate reader (SpectraMax i3x, Molecular Devices, USA).

### Subcutaneous tumor xenograft

BALB/c-nude mice were irradiated with 2.0 Gy cobalt-60 γ-ray, and subcutaneously inoculated with KG-1a or SU-DHL-6 cells (1×10^7^ cells). The length and width of tumor were measured with a vernier caliper every 5 days. The tumor volume was calculated as: tumor volume (V) = (length × width ^ 2)/2. Apoptotic gene expression levels of tumor cells were detected by qPCR. All animal procedures were approved and conducted in accordance with institutional and national animal welfare guidelines.

### Statistical analysis

GraphPad Prism 10.1.2 was used for statistical analysis. Comparisons between two groups were performed using an unpaired Student’s *t*-test when the data were normally distributed and variances were homogeneous, or the Mann-Whitney *U* test when the normality assumption was not met. For comparisons among multiple groups, one-way analysis of variance followed by Tukey’s multiple-comparisons test was used when the data were normally distributed and variances were homogeneous, whereas the Kruskal-Wallis test followed by Dunn’s multiple-comparisons test was used when the normality assumption was not met. For clinical score and body weight analyses, data from day 0 to day 6 were excluded to eliminate the interference of early irradiation-related stress. Survival was analyzed using the log-rank test. Data were presented as mean ± standard deviation. Statistical significance was established as p < 0.05.

## Results

### PF may prevent aGVHD by acting on multiple targets

**Figure 1:**
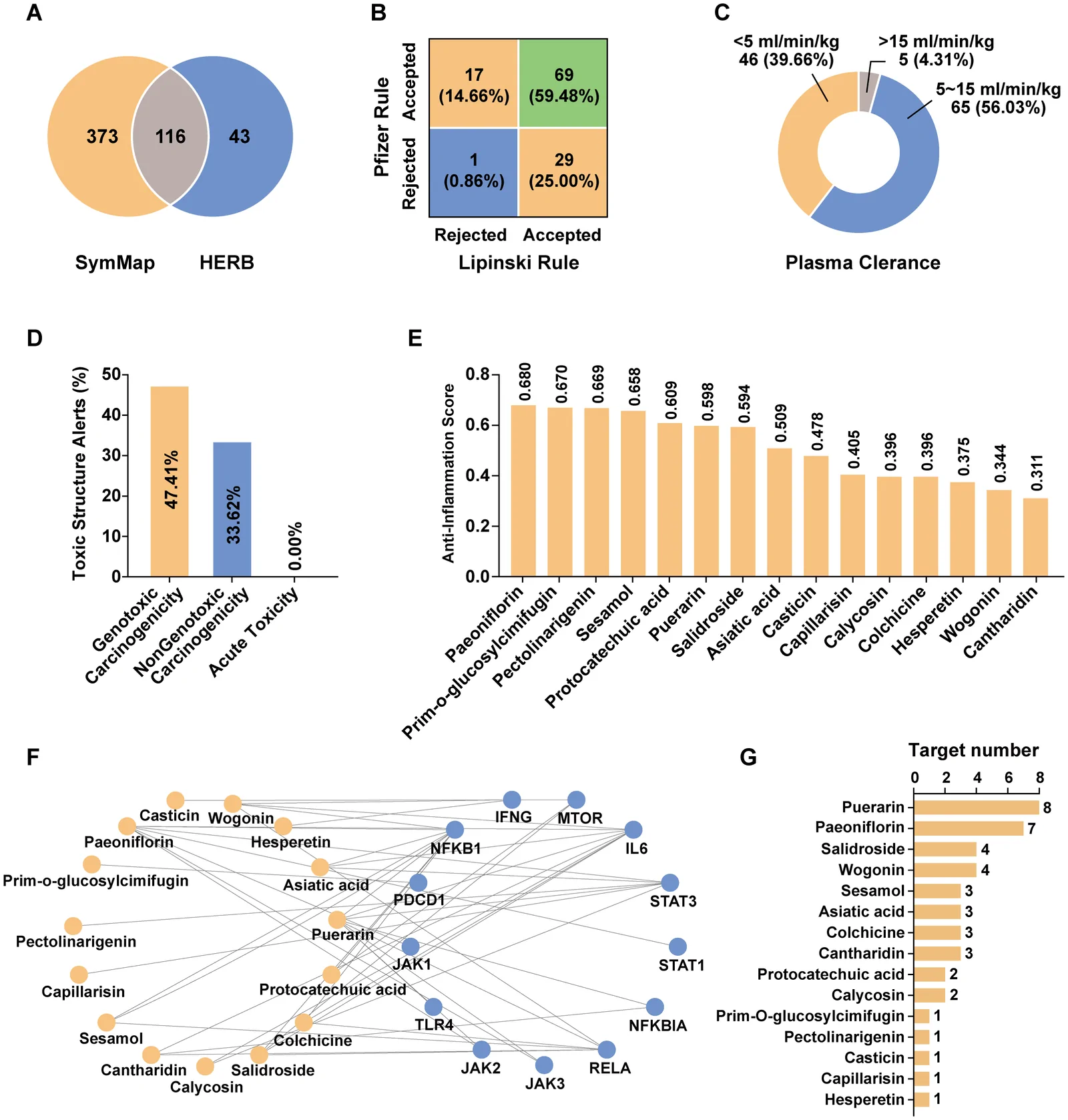
Screening of potential herbal compounds against aGVHD. **(A)** Predicting active compounds from SymMap and HERB databases against aGVHD. **(B-D)** Screening by **(B)** Lipinski/Pfizer Rule, **(C)** plasma clearance, and **(D)** structural alerts based on ADMETlab platform. **(E)** Prediction of anti-inflammatory activity of compounds based on InflamNat database. **(F)** Correlation network analysis between compounds and aGVHD key target genes. **(G)** aGVHD target numbers of compounds.

To screen the herbal bioactive compounds against aGVHD, two public databases SymMap and HERB were applied, which respectively predicted 489 and 159 unique compounds targeting aGVHD based on its relevant signaling pathway molecules (**Figure 1A**). There were a total of 116 compounds in the intersection of two databases (**Figure 1A**). Utilizing two public drug screening platforms, ADMETlab and InflamNat, we found that PF exhibited favorable pharmacokinetic property and the best anti-inflammation scores (**Figures 1B-E**). Correlation network analysis revealed that PF held one of the highest numbers of aGVHD-associated target genes, including JAK1, JAK2, JAK3, STAT1, STAT3, IL6, and NFKB1 (**Figures 1F, G**). Molecular docking confirmed the binding activities of PF to these key targets (**Supplementary Figures 1A–G**). These results suggested that PF was a promising multi-targeted compound for aGVHD prevention.

### PF effectively alleviated aGVHD and enhanced hematopoietic reconstitution

**Figure 2:**
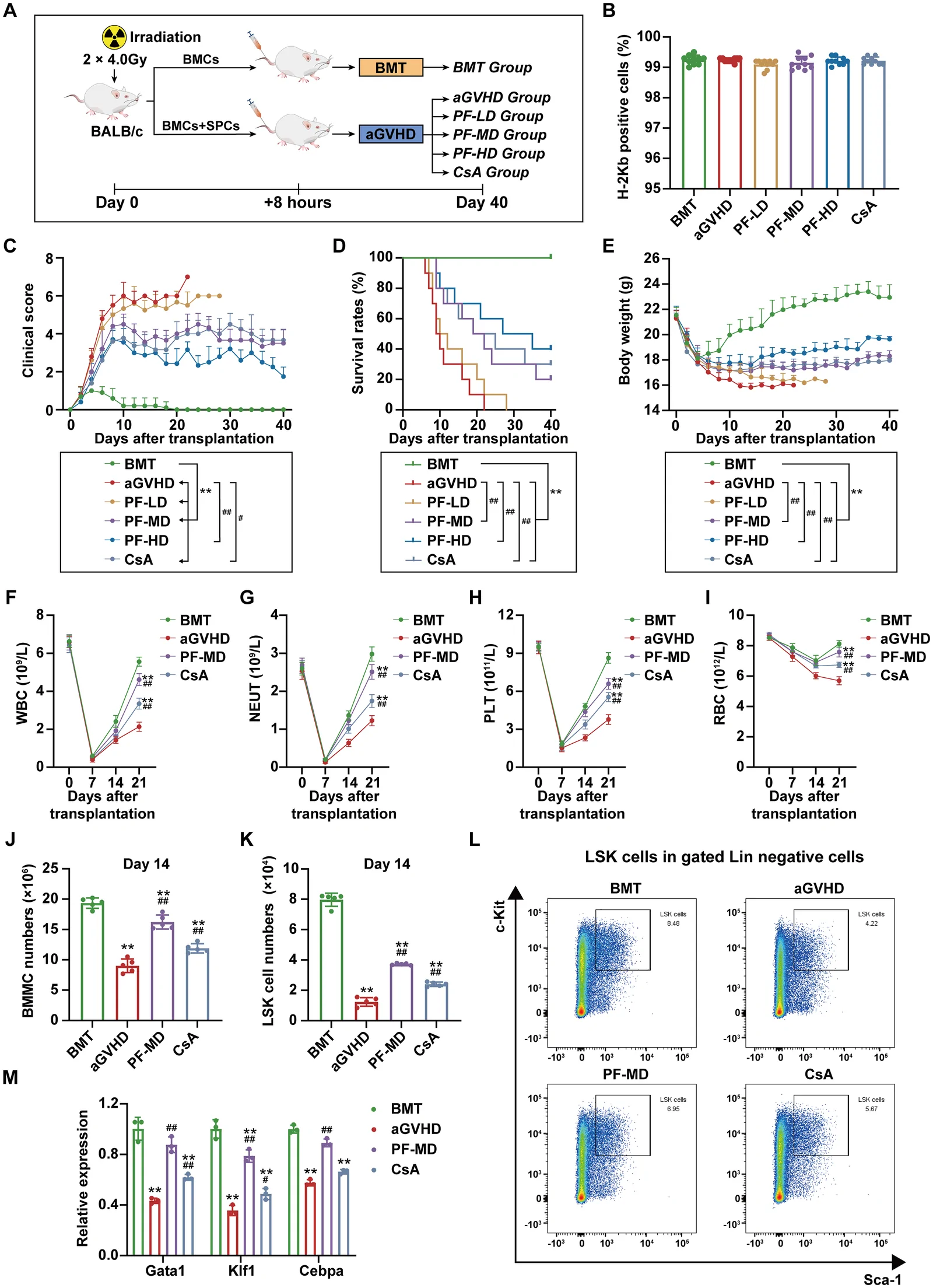
PF alleviated aGVHD and enhanced hematopoietic reconstitution in mouse model. **(A)** The schematic presentation of B6-to-BALB/c aGVHD mouse model. **(B)** The proportion of donor-derived H-2Kb^+^ cells in bone marrow was evaluated by flow cytometry at day 40 or upon mortality (n = 10). **(C-E)** The clinical scores **(C)**, survival status **(D)**, and body weight **(E)** were evaluated from day 0 to day 40 (n = 10). Data from day 0 to day 6 were excluded for statistical analysis. Intergroup comparison of clinical scores was performed using the Kruskal-Wallis test followed by Dunn’s post tests. **(F-I)** The blood cell counts, including white blood cell (WBC) **(F)**, neutrophil (NEUT) **(G)**, platelet (PLT) **(H)**, and red blood cell (RBC) **(I)** were assessed from day 0 to day 21 (n = 6). Blood cell counts were assessed in an independent cohort. **(J)** The BMMC numbers in bilateral femurs were evaluated by automated cell counter at day 14 (n = 5). **(K, L)** The HSPC populations were evaluated by flow cytometry analysis **(L)**, and absolute numbers were calculated based on BMMC numbers **(K)** at day 14 (n = 5). **(M)** The relative mRNA expression levels of *Gata1*, *Klf1*, and *Cebpa* in sorted HSPCs were evaluated using real-time qPCR at day 14 (n = 3) ^**^p < 0.01 (vs. BMT group), ^#^p < 0.05 (vs. aGVHD group), ^##^p < 0.01 (vs. aGVHD group).

To explore the prophylactic effects of PF on aGVHD, we employed two MHC mismatched aGVHD mouse models, including the classic B6-to-BALB/c model, and additional BALB/c-to-B6 model. Notably, CD3^+^ T cell proportions were approximately 30% in both B6 and BALB/c donor SPCs, indicating that there were adequate T cells inducing aGVHD occurrence after transplantation (**Supplementary Figure 2A**) (44, 45). In B6-to-BALB/c model, donor H-2Kb^+^ cells accounted for over 99.0% of bone marrow mononuclear cells (BMMCs) in each group, indicating successful engraftment of donor cells (**Figures 2A, B**). As expected, mice in aGVHD group exhibited higher clinical scores, progressive body weight loss, and increased mortality, accompanied by hunched posture, ruffled fur, and severe diarrhea, compared with BMT group, which did not exhibit these symptoms (**Figures 2C–E**). These results suggested that the aGVHD mouse model was successfully induced. Different doses of PF were administered to aGVHD mice, and the results showed that medium- and high-dose PF significantly reduced clinical scores, alleviated body weight loss, and prolonged survival (**Figures 2C–E**). In addition, similar results were observed in the BALB/c-to-B6 aGVHD model (**Supplementary Figures 2B–F**). Given that PF-MD group exhibited comparable preventive effects with CsA group, we employed 30 mg/kg/day for the subsequent experiments to avoid potential toxicity of higher doses. Our biochemistry results also showed that this dose did not induce significant hepatotoxicity or nephrotoxicity in B6-to-BALB/c transplantation (**Supplementary Table 4**).

Delayed or failed hematopoietic reconstitution represents a serious complication of aGVHD, which increases the transplant-related mortality (46). To investigate the effects of PF on hematopoiesis, we assessed blood cell counts, BMMC and HSPC populations, and the mRNA expression of hematopoietic output-associated transcription factors in HSPCs of BALB/c recipients. Results indicated that aGVHD group exhibited significantly blood cell reduction during the first 7 days after transplantation, and impaired recovery in the subsequent 2 weeks compared with BMT controls (**Figures 2F–I**). Moreover, aGVHD group showed reduced BMMC and HSPC numbers in bone marrow, accompanied by downregulated expression of transcription factors associated with hematopoietic differentiation in sorted HSPCs, including *Gata1*, *Klf1*, and *Cebpa* (**Figures 2J–M**, LSK cell gating strategy: **Supplementary Figure 2G**). Notably, PF administration substantially restored the blood cell recovery, BMMC and HSPC reduction, and downregulation of transcription factors, outperforming the CsA group (**Figures 2F–M**). Taken together, these findings supported the ability of PF to alleviate the hematopoietic impairment associated with aGVHD.

### PF improved graft hematopoietic potential

**Figure 3:**
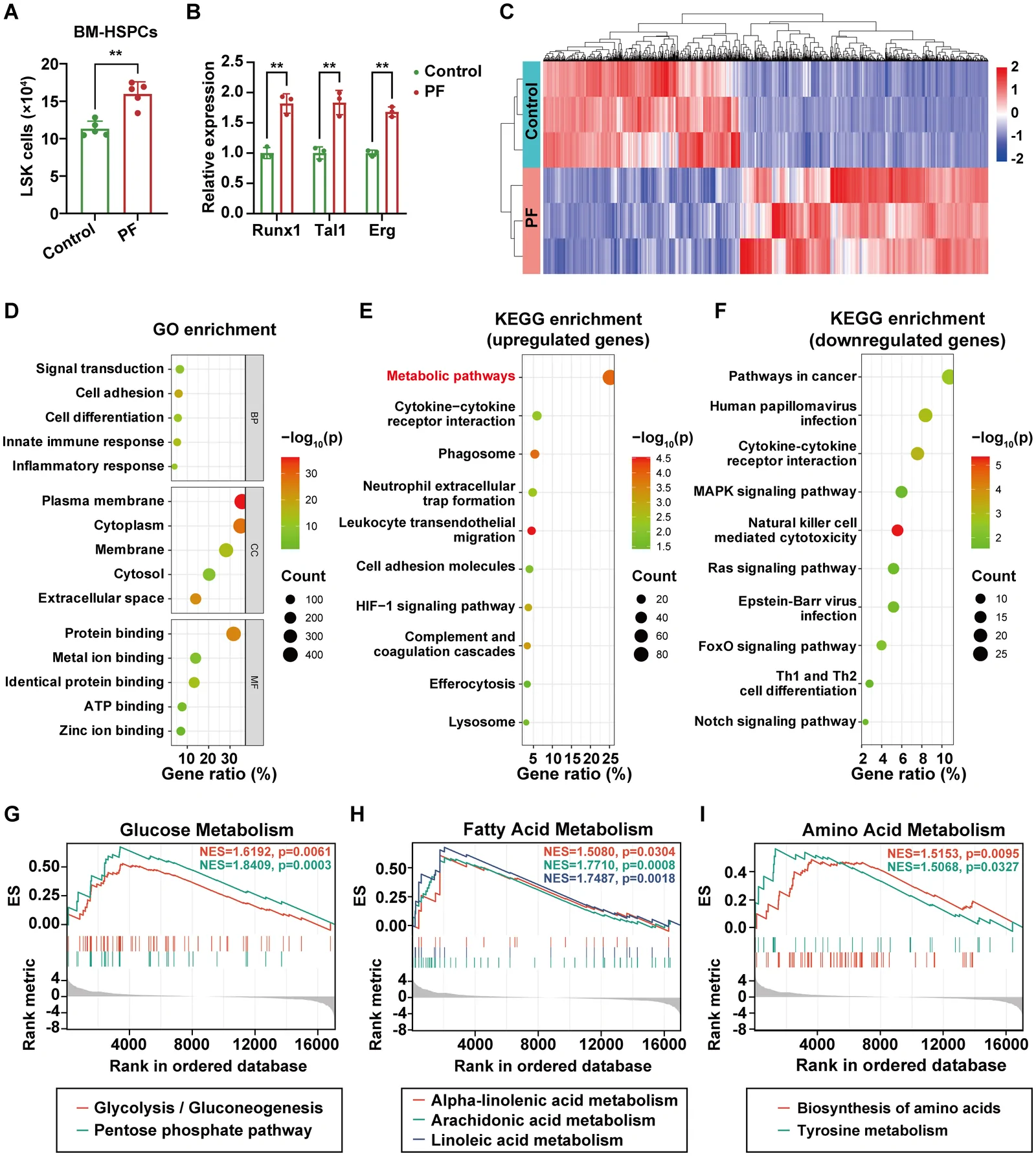
PF improved graft hematopoietic potential. **(A)** The HSPC numbers in bone marrow of B6 donor mice (n = 5). **(B)** The mRNA expression levels of *Runx1*, *Tal1*, and *Erg* in sorted HSPCs of B6 donors (n = 3). **(C)** Heat map of differentially expressed genes in HSPCs from PF-treated donor mice. **(D)** GO enrichment analysis of differentially expressed genes. **(E, F)** KEGG enrichment analysis of **(E)** upregulated genes and **(F)** downregulated genes. **(G-I)** GSEA analysis of gene sets related to **(G)** glucose metabolism pathways, **(H)** fatty acid metabolism pathways, and **(I)** amino acid metabolism pathways. ^**^p < 0.01.

The efficiency of hematopoietic reconstitution after transplantation depends not only on the recipient bone marrow niche, but also on the abundance and intrinsic functional state of donor HSPCs within the graft. Since PF substantially improved post-transplantation hematopoietic recovery, we next investigated whether pre-administration of B6 donor mice with PF could enhance the hematopoietic potential of the graft. Thus, donor mice were administered with PF for 7 days, followed by quantification, isolation, qPCR assay and sequencing analysis of bone marrow HSPCs. Results showed that PF administration significantly increased HSPC numbers in bone marrow (**Figure 3A**). In addition, PF significantly increased the mRNA expression of *Runx1*, *Tal1*, and *Erg*, the key transcription factors involved in the maintenance, self-renewal, and lineage differentiation of HSPCs (**Figure 3B**). Transcriptome sequencing revealed that a total of 1301 genes were dysregulated in HSPCs of bone marrow, with 725 genes upregulated and 576 genes downregulated (**Figure 3C**). To better elucidate the biological functions of these DEGs, we conducted GO enrichment analysis. The top 5 significantly enriched terms in biological process (BP) included signal transduction, cell adhesion, cell differentiation, innate immune response, and inflammatory response (**Figure 3D**). For cellular component (CC), altered genes were mainly enriched in plasma membrane, cytoplasm, membrane, cytosol, and extracellular space (**Figure 3D**). Additionally, the molecular function (MF) analysis showed predominant enrichment in protein binding, metal ion binding, identical protein binding, adenosine triphosphate (ATP) binding, and zinc ion binding (**Figure 3D**). KEGG analysis revealed upregulated genes significantly enriched in metabolic pathways, cytokine-cytokine receptor interaction, phagosome, hypoxia-inducible factor 1 (HIF-1) signaling pathway, lysosome and so on, which played important roles in regulating HSPCs activation, proliferation, and mobilization (47–49) (**Figure 3E**). Downregulated genes were mainly enriched in pathways in cancer, mitogen-activated protein kinase (MAPK) signaling pathway, Ras signaling pathway, forkhead box O (FoxO) signaling pathway, Notch signaling pathway and so on, which might contribute to HSPCs depletion and quiescence, as well as leukemogenesis (50–53) (**Figure 3F**).

Interestingly, we noticed that up to 12.94% of the upregulated genes were enriched in the term of metabolic pathways. Further GSEA analysis revealed that PF administration significantly upregulated glucose metabolism (glycolysis/gluconeogenesis and pentose phosphate pathway), fatty acid metabolism (arachidonic acid metabolism and linoleic acid metabolism), and amino acid metabolism (the biosynthesis of amino acids and tyrosine metabolism) (**Figures 3G–I**). Enriched core genes of each metabolic pathway were shown in the **Supplementary Table 5**. Overall, these results indicated that PF could positively regulate HSPCs, thereby improving graft hematopoietic potential.

### Entire-process PF administration significantly enhanced the efficacy of aGVHD prevention

**Figure 4:**
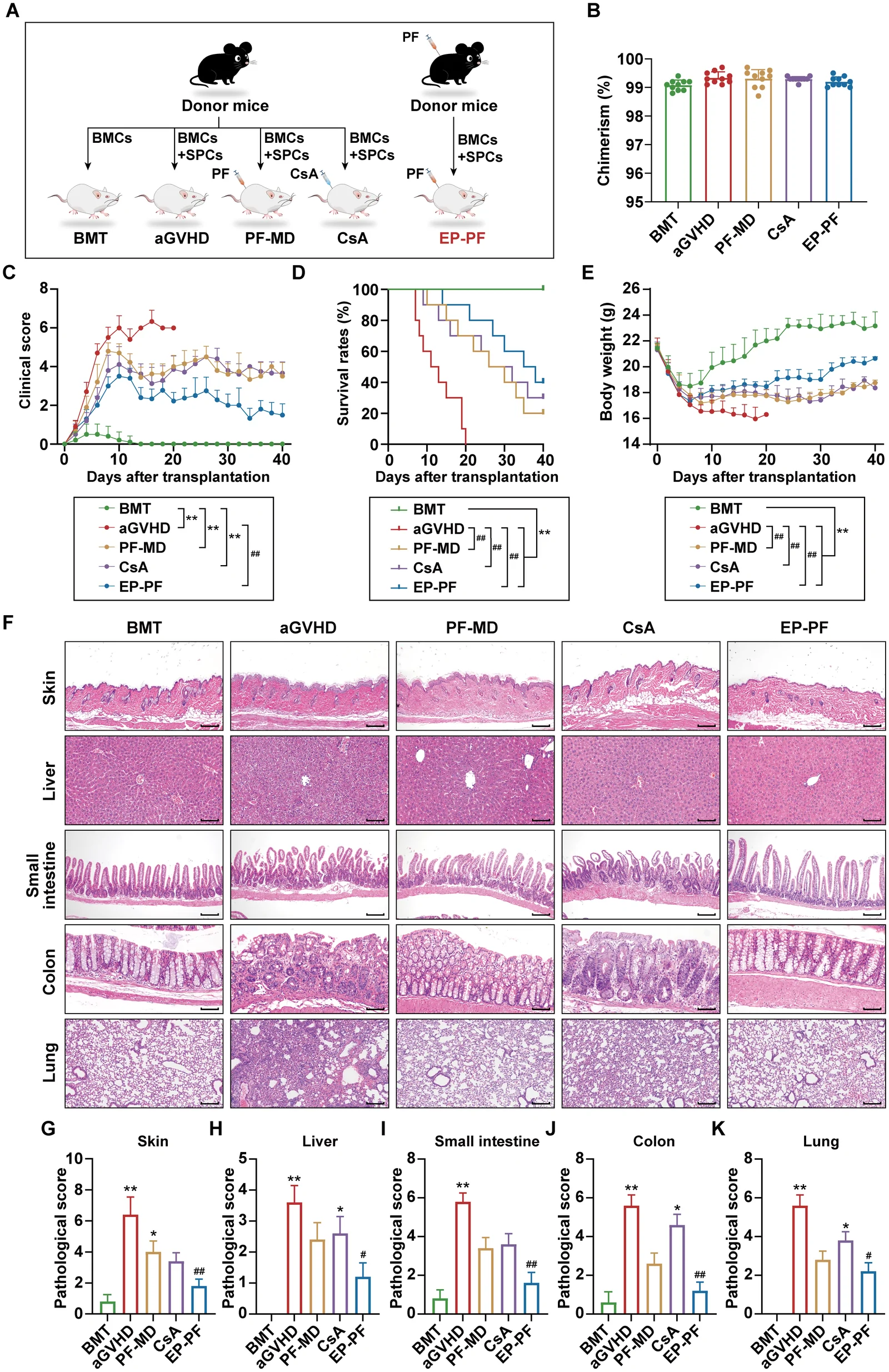
Entire-process PF administration enhanced the efficacy of aGVHD prevention. **(A)** Schematic presentation of drug administration regimen. **(B)** Surface H-2Kb positive cells from bone marrow were detected by flow cytometry at day 14 (n = 10). **(C–E)** The **(C)** clinical scores, **(D)** survival and **(E)** body weight in each group were illustrated from day 0 to day 40 (n = 10). Data from day 0 to day 6 were excluded for statistical analysis. Intergroup comparison of clinical scores was performed using the Kruskal-Wallis test followed by Dunn’s post tests. **(F)** H&E staining images of skin, liver, small intestine, colon, and lung in each group at day 14. Scale bar: 200 μm in skin, small intestine, and lung; 100 μm in liver and colon. **(G–K)** The pathological scores of **(G)** skin, **(H)** liver, **(I)** small intestine, **(J)** colon, and **(K)** lung showing the severity of aGVHD (n = 5). Intergroup comparison of pathological scores was performed using the Kruskal-Wallis test followed by Dunn’s post tests. *p < 0.05 (vs. BMT group), **p < 0.01 (vs. BMT group), ^#^p < 0.05 (vs. aGVHD group), ^##^p < 0.01 (vs. aGVHD group).

In light of the multiple roles of PF in pre- and post-transplant, we set up we set up an EP-PF group with PF administration in both B6 donors and BALB/c recipients, to improve post-transplantation hematopoietic reconstitution and the efficacy of aGVHD prevention (**Figure 4A**). H-2Kb positive cell analysis indicated successful engraftment of donor cells in each group (**Figure 4B**). We found that mice in EP-PF group exhibited superior aGVHD prophylactic efficacy compared to PF-MD and CsA groups, manifested by significantly milder clinical symptoms, less weight loss and more prolonged survival (**Figures 4C–E**). In addition, aGVHD group exhibited marked pathological injury in examined tissues, including stratum corneum thickening in the skin; disorganization of hepatic lobules with focal necrosis in the liver; villus shortening, blunting, and separation in the small intestine; crypt destruction and goblet cell loss in the colon; and alveolar septal thickening, disruption of alveolar architecture, and inflammatory cell infiltration in the lung (**Figure 4F**). PF-MD, CsA, and EP-PF attenuated these abnormalities to varying degrees (**Figure 4F**). Of note, EP-PF group showed the most significant reversal of damage, characterized by stratum corneum normalization in the skin, hepatic lobule integrity in the liver, gut villus alignment in the small intestine, goblet cell recovery in the colon, as well as inflammatory cell reduction in above organs (**Figure 4F**). Consistently, pathological scores were significantly higher in the aGVHD group than in the BMT group in all five examined tissues, whereas EP-PF significantly reduced these scores, with the greatest overall improvement among the administration groups (**Figures 4G–K**).

### PF reduced donor T cell infiltration and rescued gene expression dysregulation

**Figure 5:**
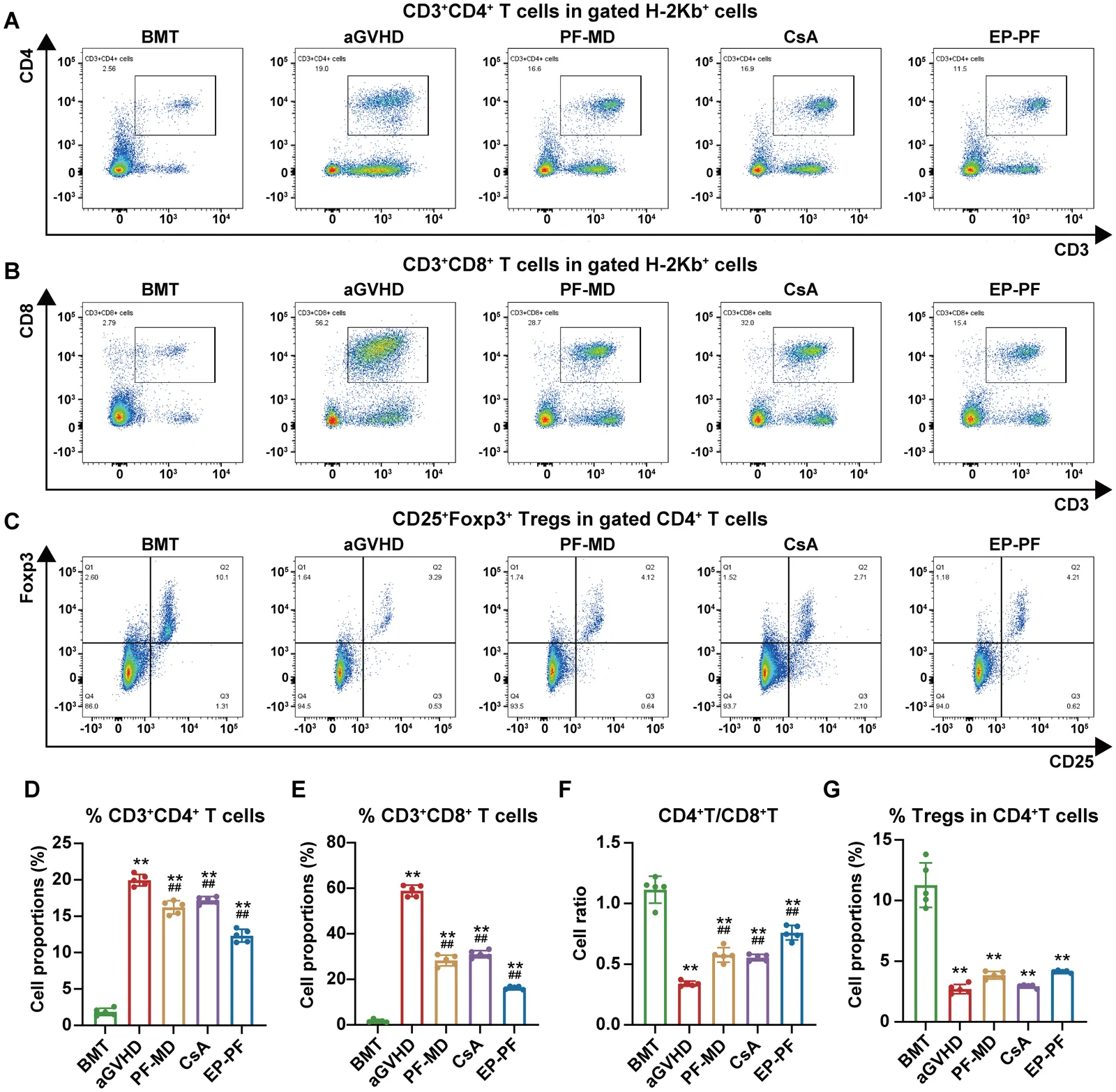
PF reduced donor T cell infiltration in gut. **(A–C)** The intestinal representative flow cytometry plots of **(A)** CD3^+^CD4^+^ T cells in gated H-2Kb^+^ cells, **(B)** CD3^+^CD8^+^ T cells in gated H-2Kb^+^ cells, and **(C)** CD25^+^Foxp3^+^ Tregs in gated CD4^+^ T cells at day 14. **(D, E)** The proportions of **(D)** CD3^+^CD4^+^ T cells and **(E)** CD3^+^CD8^+^ T cells in each group at day 14 (n = 5). **(F)** The CD4^+^/CD8^+^ T cell ratio in each group at day 14 (n = 5). **(G)** The proportions of CD25^+^Foxp3^+^ Tregs in each group at day 14 (n = 5). **p < 0.01 (vs. BMT group), ^##^p < 0.01 (vs. aGVHD group).

Donor T cell infiltration in target tissues represents a major pathological characteristic of aGVHD, driving severe inflammation and tissue injury. To assess the infiltration of donor T cells, we analyzed the numbers and proportions of donor CD4^+^ T and CD8^+^ T cells in recipient gut at 14 days after transplantation using flow cytometry (T cell gating strategy: **Supplementary Figure 3A**). Our results showed that the aGVHD group exhibited significantly increased numbers and proportions of donor-derived CD4^+^ T and CD8^+^ T cells in the gut compared with BMT group, indicating marked accumulation of alloreactive T cells in the target tissue (**Figures 5A, B, D, E**; **Supplementary Figures 3B, C**). The CD4^+^/CD8^+^ T cell ratio was also significantly reduced in aGVHD, suggesting a relative predominance of CD8^+^ T cell infiltration (**Figure 5F**). Notably, PF administration significantly decreased the cell numbers and proportions of both CD4^+^ T and CD8^+^ T cells derived from donor mice, and restored the CD4^+^/CD8^+^ T cell balance (**Figures 5A, B, D–F**; **Supplementary Figures 3B, C**). Given the potential protective roles of immunoregulatory cells, we further examined whether PF affected the Treg and MDSC populations in the gut. However, results showed that PF administration did not significantly alter the proportions of CD25^+^Foxp3^+^ Tregs and CD11b^+^Gr-1^+^ MDSCs (**Figures 5C, G**; **Supplementary Figure 3D–F**).

**Figure 6:**
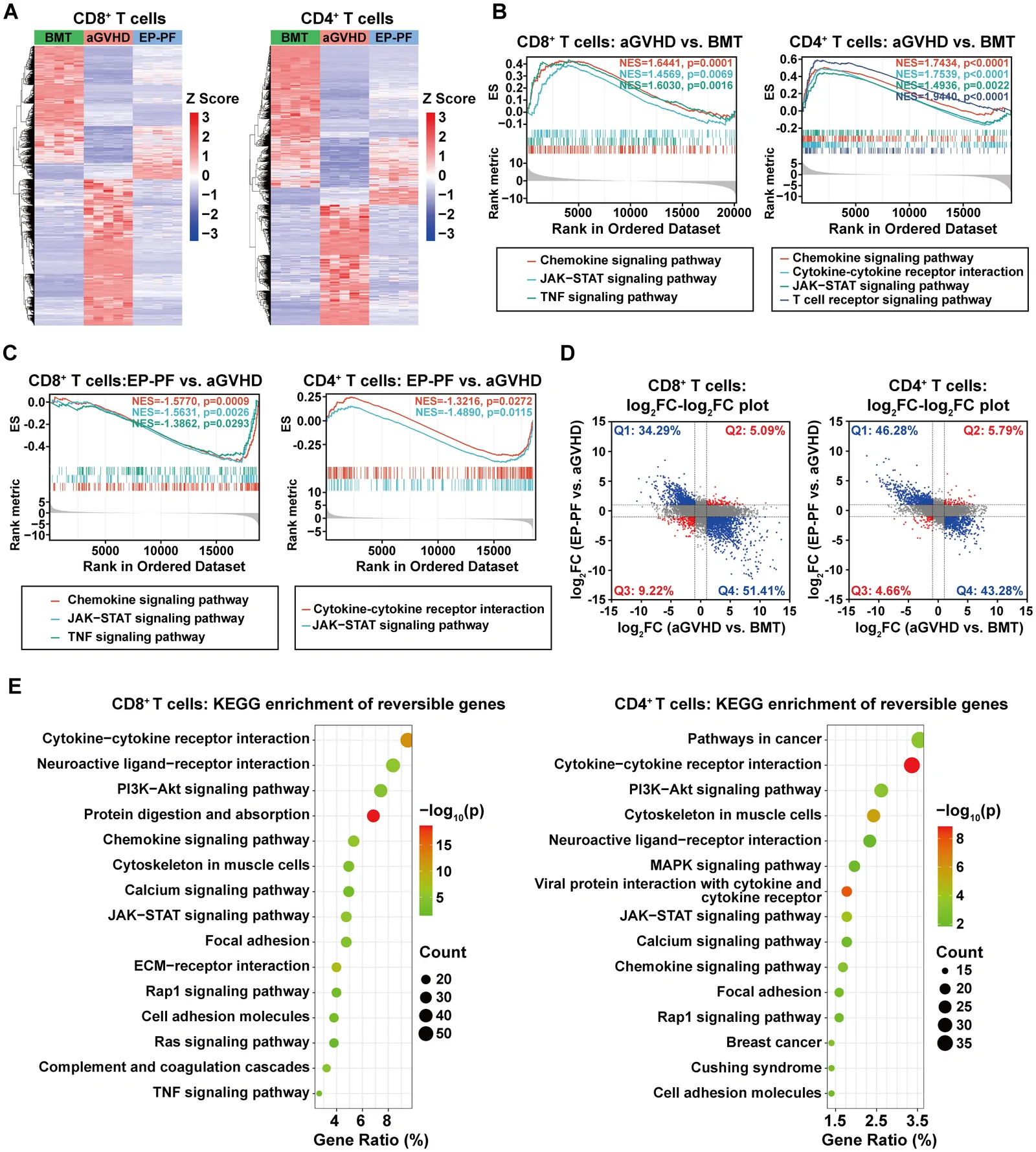
PF rescued gene expression dysregulation in splenic donor T cells. **(A)** Gene expression heatmap of DEGs in donor CD8^+^ T cells and CD4^+^ T cells. **(B)** GSEA analysis of donor CD8^+^ T cells and CD4^+^ T cells in aGVHD vs. BMT comparison. **(C)** GSEA analysis of donor CD8^+^ T cells and CD4^+^ T cells in EP-PF vs. aGVHD comparison. **(D)** EP-PF restored the abnormal gene expression patterns of donor CD8^+^ T cells and CD4^+^ T cells in aGVHD group. Genes in Q1 zone (decreased in aGVHD group but increased in EP-PF group) and Q4 zone (increased in aGVHD group but decreased in EP-PF group) were defined as reversible genes. Genes were considered fully restored when |log_2_[FC (EP-PF vs. BMT)]| < 1. **(E)** KEGG enrichment of reversible genes in donor CD8^+^ T cells and CD4^+^ T cells. NES: normalized enrichment score.

To further investigate the functional alterations of donor T cells, we performed the RNA sequencing (RNA-Seq) analysis to examine gene expression profiles in splenic donor T cells. Compared with BMT group, there were 9,247 DEGs in CD8^+^ T cells, including 4,548 upregulated genes and 4,699 downregulated genes (**Figure 6A**). KEGG and GSEA analyses indicated that aGVHD-associated signaling pathways were obviously upregulated, especially chemokine signaling pathway, JAK-STAT signaling pathway, and TNF signaling pathway (**Figure 6B**; **Supplementary Figure 4A**). These results again demonstrated the successful construction of aGVHD model. Notably, we found that EP-PF administration could partly reverse the gene profile alterations caused by aGVHD, with 2,591 genes differentially expressed following EP-PF administration (**Figures 6A, C**; **Supplementary Figure 4A**). The log_2_FC-log_2_FC plot showed that EP-PF rectified 85.70% of intersected abnormal genes toward BMT expression levels, with 38.25% fully restored and 61.75% partially restored (**Figure 6D**). Moreover, KEGG enrichment analysis identified that chemokine signaling pathway, JAK-STAT signaling pathway and TNF signaling pathway were also significantly enriched in these reversible genes (**Figure 6E**).

As for donor CD4^+^ T cells of aGVHD group, there were 2,026 upregulated genes and 2,725 downregulated genes in comparison to BMT group (**Figure 6A**). KEGG and GSEA analysis revealed significant activation of chemokine signaling pathway, cytokine-cytokine receptor interaction, JAK-STAT signaling pathway, and T cell receptor signaling pathway, which were highly associated with aGVHD progress (**Figure 6B**; **Supplementary Figure 4B**). These results indicated robust inflammatory signaling activation in CD4^+^ T cells of aGVHD group. However, EP-PF administration obviously reduced pro-inflammatory signaling pathways of CD4^+^ T cells (**Figures 6A, C**; **Supplementary Figure 4B**). Similarly, EP-PF adjusted 89.56% of intersected dysregulated genes toward the expression pattern of BMT group, with 45.22% completely restored and 54.78% partially restored (**Figure 6D**). Indeed, these reversible genes were also enriched in cytokine-cytokine receptor interaction and JAK-STAT signaling pathway (**Figure 6E**).

### PF recovered the balance of cytokines and chemokines

**Figure 7:**
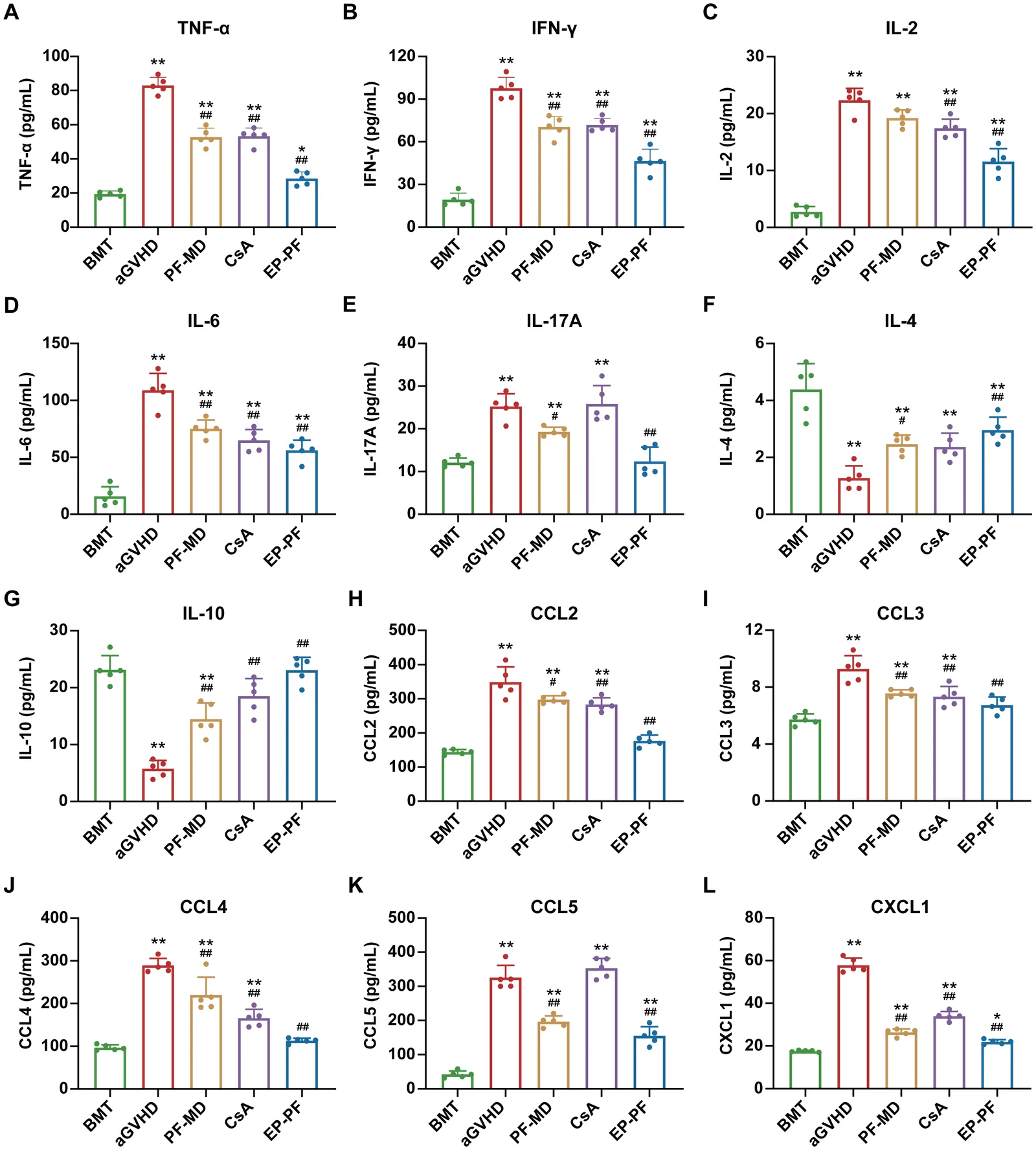
PF recovered the balance of cytokines and chemokines. **(A–E)** PF decreased the serum levels of pro-inflammatory cytokines, including **(A)** TNF-α (n = 5), **(B)** IFN-γ (n = 5), **(C)** IL-2 (n = 5), **(D)** IL-6 (n = 5) and **(E)** IL-17A at day 14 (n = 5). **(F, G)** PF increased the serum levels of anti-inflammatory cytokines, including **(F)** IL-4 (n = 5) and **(G)** IL-10 at day 14 (n = 5). **(H-L)** PF inhibited the release of **(H)** CCL2 (n = 5), **(I)** CCL3 (n = 5), **(J)** CCL4 (n = 5), **(K)** CCL5 (n = 5) and **(L)** CXCL1 at day 14 (n = 5). *p < 0.05 (vs. BMT group), **p < 0.01 (vs. BMT group), ^#^p < 0.05 (vs. aGVHD group), ^##^p < 0.01 (vs. aGVHD group).

aGVHD is accompanied by severe inflammatory responses, characterized by uncontrolled release of pro-inflammatory cytokines, a disbalance of chemokines and their receptors, abnormal activation of alloreactive T cells, and subsequent tissue damage. Results mentioned above reflected this fact, while the cytokine-cytokine receptor interaction, inflammatory signaling pathways and chemokine signaling pathways in donor T cells were significantly activated. To further investigate whether PF affected the production of cytokines and chemokines, we tested the serum levels of multiple proteins associated with aGVHD. Results showed that aGVHD group exhibited elevated levels of pro-inflammatory cytokines, including TNF-α, IFN-γ, IL-2, IL-6 and IL-17A, while PF and CsA administration could significantly reduce their levels (**Figures 7A–E**). Notably, EP-PF group showed the best inhibitory effects (**Figures 7A–E**). Meanwhile, results revealed that IL-4 and IL-10, which were associated with T helper 2 (Th2) and immunoregulatory responses, were significantly reduced in aGVHD mice, and PF obviously increased their serum levels, with most pronounced in EP-PF group (**Figures 7F, G**). Moreover, multiple chemokines, including CCL2, CCL3, CCL4, CCL5, and CXCL1, were remarkably elevated in aGVHD mice and may contribute to the recruitment of inflammatory cells to target organs (**Figures 7H–L**). However, PF, CsA and especially EP-PF administration significantly reversed the overexpression of chemokines (**Figures 7H–L**). These results suggested that PF could effectively inhibit the release of inflammatory cytokines and chemokines, and elevate the anti-inflammatory cytokine levels, thereby inhibiting the immune response mediated by aGVHD.

### PF alleviated aGVHD-induced gut damage and balanced the gut microbiota

**Figure 8:**
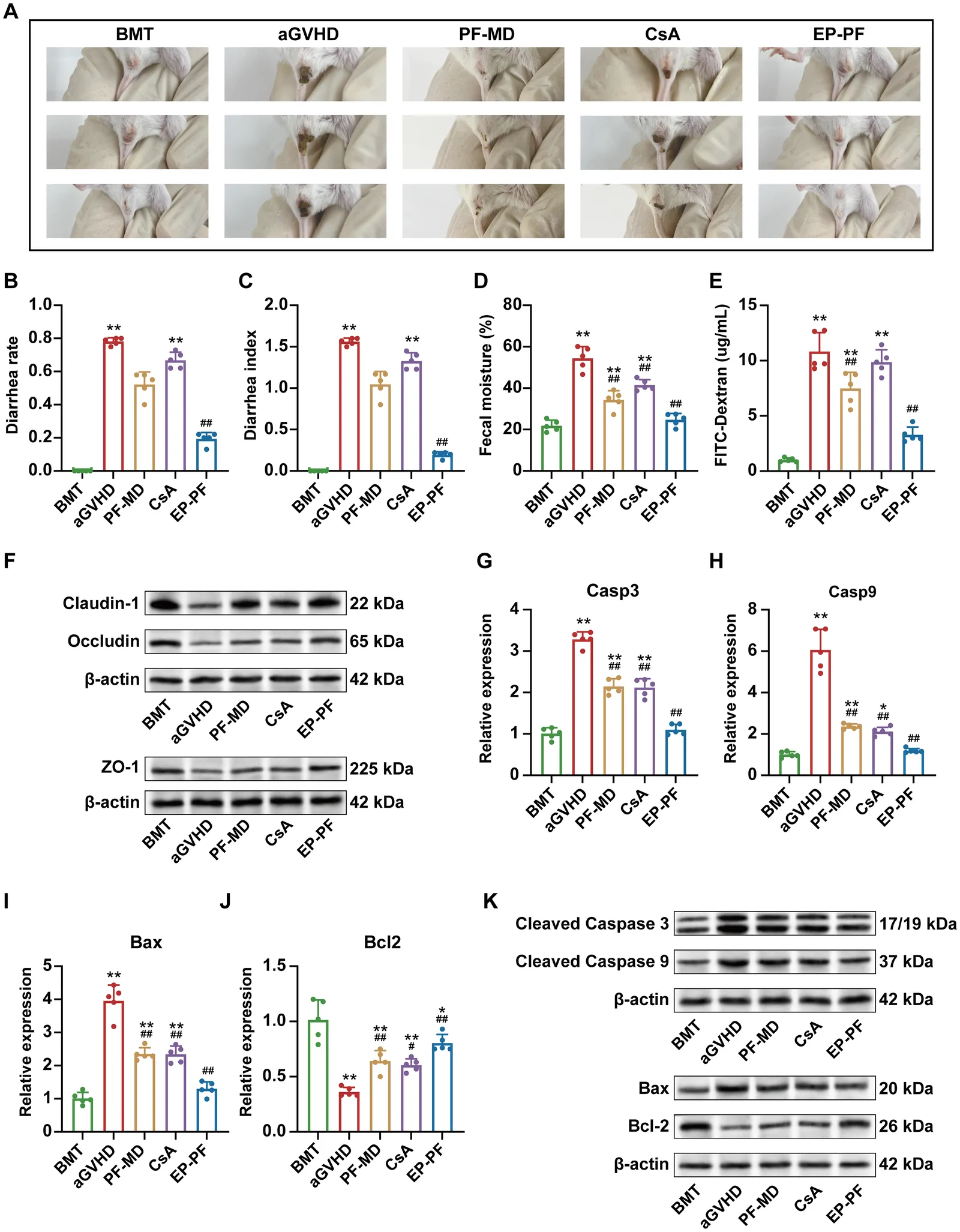
PF alleviated aGVHD-induced gut damage. **(A)** Representative images of diarrhea in each group. Diarrhea was characterized by fecal contamination in the anus of the mice. **(B–D)** PF decreased the **(B)** diarrhea rate, **(C)** diarrhea index, and **(D)** fecal moisture in aGVHD mice at 14 days after transplantation (n = 5). Intergroup comparisons of diarrhea rate and diarrhea index were performed using the Kruskal-Wallis test followed by Dunn’s post tests. **(E)** PF recovered the gut permeability in aGVHD mice, demonstrated by FITC-Dextran assay (n = 5). **(F)** PF recovered the expression of intestinal tight junction proteins, including Claudin-1, Occludin, and ZO-1. **(G–J)** PF downregulated apoptotic genes, including **(G)** Casp3, **(H)** Casp9, and **(I)** Bax, while upregulated anti-apoptotic gene **(J)** Bcl2 (n = 5). **(K)** PF decreased apoptotic proteins, including cleaved caspase 3, cleaved caspase 9, and Bax, while increased anti-apoptotic protein Bcl-2. *p < 0.05 (vs. BMT group), **p < 0.01 (vs. BMT group), ^#^p < 0.05 (vs. aGVHD group), ^##^p < 0.01 (vs. aGVHD group).

Gut damage, the leading cause of mortality in aGVHD patients, seriously impacts the long-term survival of patients, commonly characterized by diarrhea (54). Our results showed that aGVHD mice developed severe diarrhea at 14 days after transplantation, with significantly increased diarrhea rates, diarrhea index and fecal moisture (**Figures 8A–D**). However, PF or CsA could significantly alleviate diarrhea symptoms, particularly in the EP-PF group (**Figures 8A–D**). In addition, the FITC-Dextran assay showed that intestinal permeability was significantly increased in aGVHD mice, while Western blotting revealed markedly reduced levels of the tight junction proteins Claudin-1, Occludin, and ZO-1, together indicating severe gut barrier injury induced by aGVHD (**Figures 8E, F**). Similarly, these alterations could be restored by PF and CsA, especially in the EP-PF group (**Figures 8E, F**). Not only that, intestinal tissues from aGVHD mice exhibited increased mRNA and protein expression of the apoptosis-related genes Casp3, Casp9, and Bax, accompanied by reduced expression of the anti-apoptotic gene Bcl2 (**Figures 8G–K**). Similarly, PF and CsA administration effectively reversed these molecular abnormalities, especially in the EP-PF group (**Figures 8G–K**).

**Figure 9:**
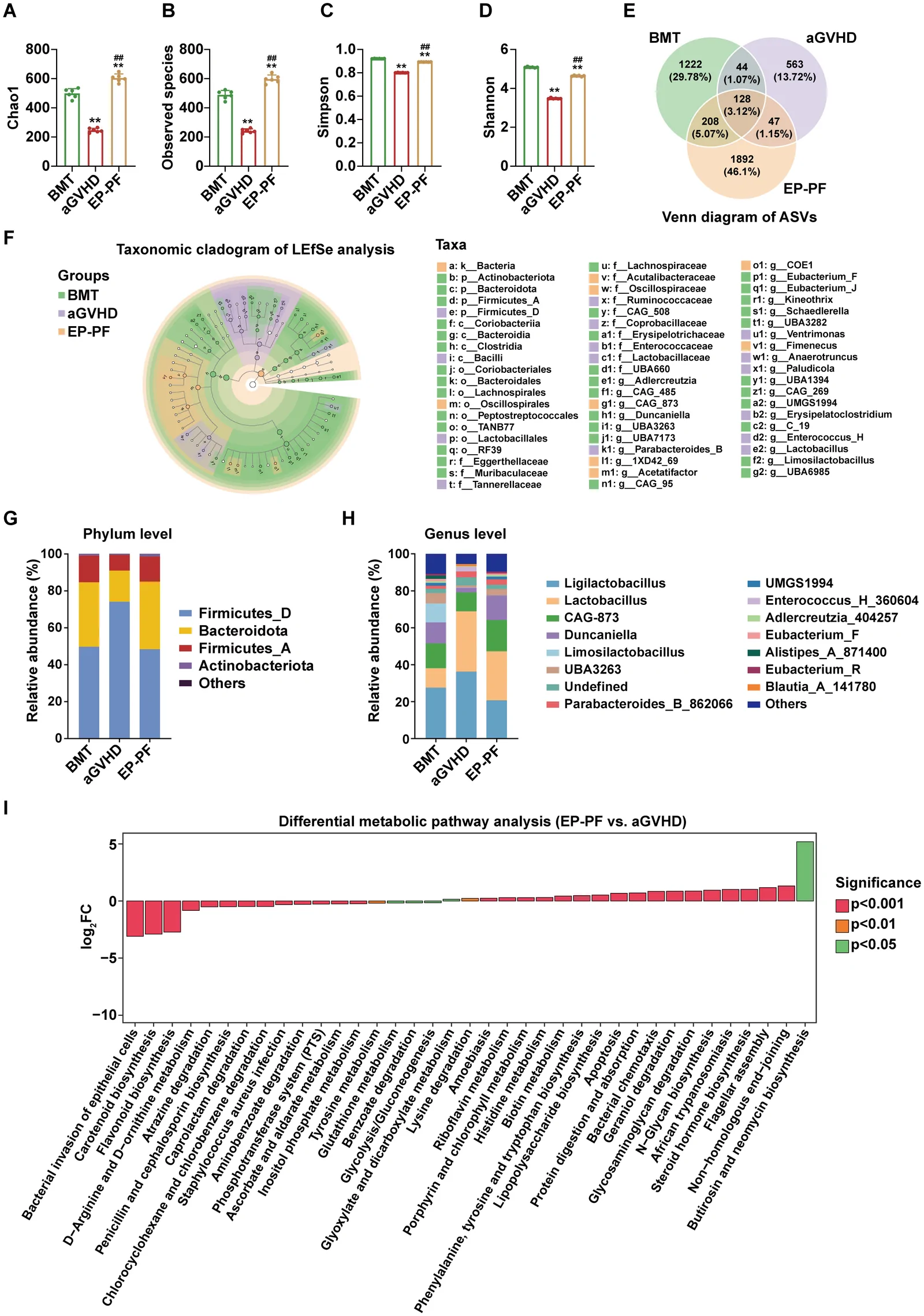
PF balanced the gut microbiota in aGVHD. **(A–D)** PF increased the gut microbial diversity, based on **(A)** Chao1, **(B)** Observed species, **(C)** Simpson, and **(D)** Shannon (n = 6). **(E)** Overall ecological patterns were analyzed by Venn diagram. **(F)** LEfSe analysis identified biomarker taxa of each group (linear discriminant analysis score > 2, p < 0.05). **(G, H)** Structure of microbial composition in each group at the **(G)** phylum and **(H)** genus levels. **(I)** Differential metabolic pathways following EP-PF administration. **p < 0.01 (vs. BMT group), ^##^p < 0.01 (vs. aGVHD group).

In addition, diarrhea is closely associated with gut microbiota dysbiosis, and a series of studies reported that gut dysbiosis occurs in aGVHD (55–57). Our results showed that the gut microbiota of aGVHD group was significantly decreased in diversity and changed in composition (**Figures 9A–H**). The amplicon sequence variants (ASVs) of gut microbiota between aGVHD and BMT group showed different patterns (**Figure 9E**). Subsequent LEfSe analysis identified 15 biomarker taxa of aGVHD group across taxonomic levels (linear discriminant analysis score > 2, p < 0.05), such as *Lactobacillus*, *Lactobacillales*, *Bacilli* and *Firmicutes_D*, which were significantly different from that of BMT group (like *Muribaculaceae*, *Bacteroidota*, *Bacteroidales*) (**Figure 9F**). At the phylum level, aGVHD was characterized by significant *Firmicutes_D* expansion, as well as *Bacteroidota* and *Firmicutes_A* reduction (**Figure 9G**). At the genus level, *Lactobacillus* expanded in aGVHD mice, while *Duncaniella* and *Limosilactobacillus* were decreased (**Figure 9H**). Targeting these abnormal species and restoring gut microbiota balance may provide novel therapeutic strategies for aGVHD patients and improve post-transplant prognosis (56, 57).

Previous studies demonstrated that PF could restore healthy microbial composition of Bacteroidota, Firmicutes and others in ulcerative colitis and diabetic cardiomyopathy, indicating the benefits of paeoniflorin in gut microbial balance (29, 30). However, studies investigating the effects of PF on the gut microbiota in aGVHD remain lacking. Our study showed that EP-PF administration could substantially recover the microbial diversity in aGVHD (**Figures 9A–D**). Following EP-PF administration, characteristic taxa shifted to 9 taxa such as *CAG_873*, *1XD42_69*, *COE1*, and *Acutalibacteraceae*, distinct from the aGVHD group (**Figure 9F**). Concretely, EP-PF administration reversed the percentages of *Firmicutes_D*, *Bacteroidota* and *Firmicutes_A* at the phylum level, and the abundance of *Lactobacillus* and *Duncaniella* at the genus level (**Figures 9G, H**). Subsequently, we focused on the biological relevance of these compositional alterations following EP-PF administration, and conducted a differential metabolic pathway analysis. Our results showed that the term butirosin and neomycin biosynthesis was significantly upregulated, and the term bacterial invasion of epithelial cells was obviously downregulated in EP-PF group, and we suspected that EP-PF may help protect gut epithelial cells against pathogens by augmenting antibiotic-synthesizing microbiota (**Figure 9I**).

### PF preserved graft-versus-tumor effects

**Figure 10:**
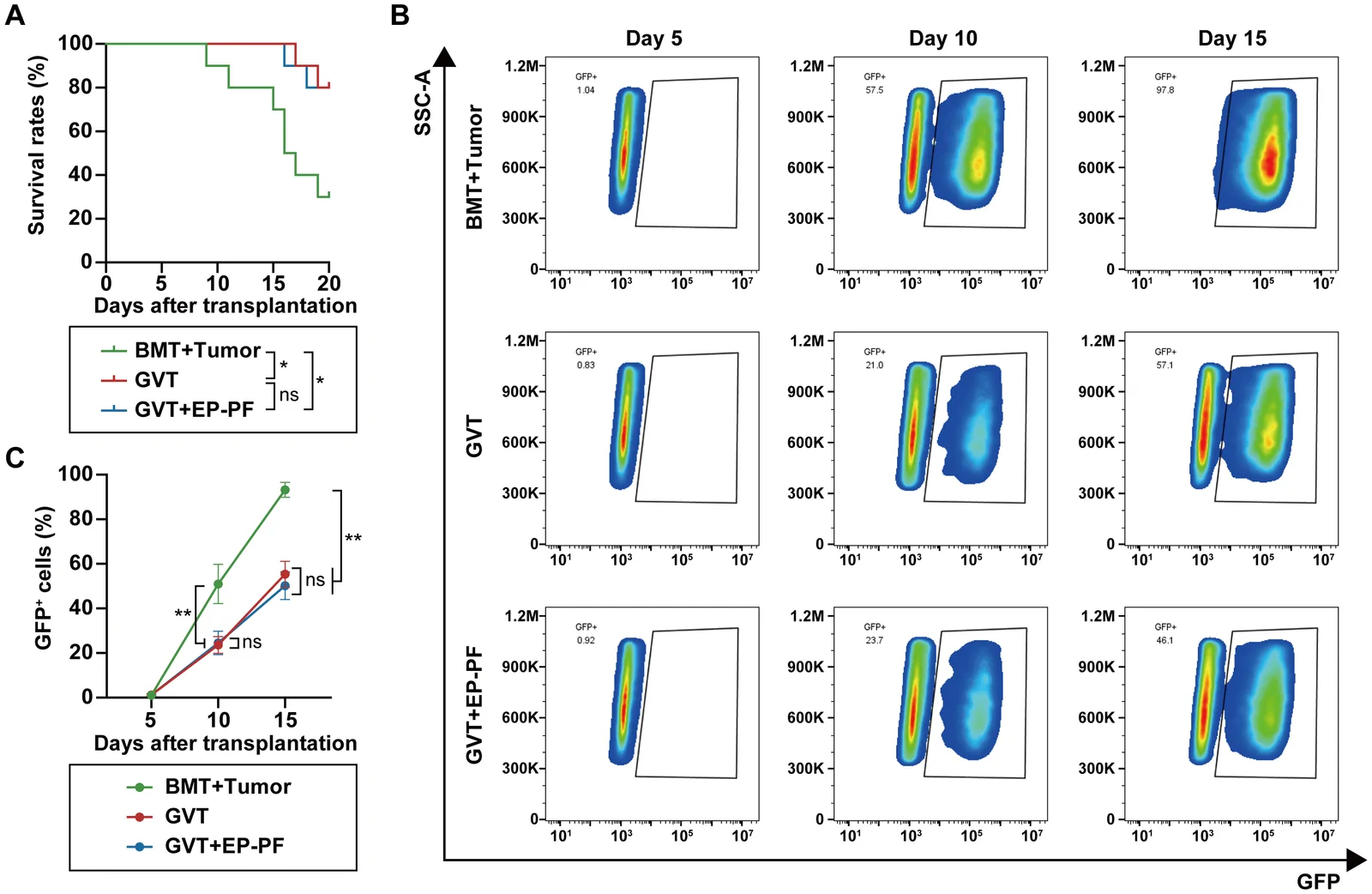
PF preserved graft-versus-tumor effects. BALB/c recipient mice underwent allogeneic bone marrow transplantation and were simultaneously inoculated with GFP-labeled A20 murine B-cell lymphoma cells. Mice in the GVT and GVT+EP-PF groups additionally received donor splenocytes, while mice in the GVT+EP-PF group received entire-process paeoniflorin administration. **(A)** Survival curves of mice in the BMT+Tumor, GVT, and GVT+EP-PF groups from day 0 to day 20 after transplantation (n = 10). **(B)** Representative flow cytometry plots showing the GFP^+^ A20 cell proportions in peripheral blood at day 5, day 10, and day 15 after transplantation. **(C)** Quantification of GFP^+^ A20 cell proportions in peripheral blood at day 5, day 10, and day 15 after transplantation (BMT+Tumor group: n = 10, 9, 7 at day 5, 10, 15; GVT and GVT+EP-PF groups: n = 10, 10, 10 at day 5, 10, 15). *p < 0.05, **p < 0.01, ns, not significant.

The relapse of primary hematological malignancies also represents a crucial cause of transplant failure (1). Commonly, immunosuppressive drugs that inhibit T cell activity may impair the GVT effects and potentially increase the risk of malignant disease relapse (58). To investigate whether PF affected the GVT effect, we constructed the GVT mouse model by co-transplanting GFP-labeled A20 cells, a BALB/c-derived murine B cell lymphoma cell line. Our results showed that 70% of the mice in BMT+Tumor group died within 20 days after transplantation (**Figure 10A**). The proportion of GFP^+^ cells in peripheral blood increased rapidly and exceeded 90% on day 15 (**Figures 10B, C**). In contrast, survival was significantly prolonged in the GVT group, with 80% of the mice remaining alive on day 20 (**Figure 10A**). The proportion of circulating GFP^+^ cells was also significantly reduced, indicating robust GVT effects (**Figures 10B, C**). Notably, no significant differences in survival and tumor burden were observed between the GVT+EP-PF and GVT groups, suggesting that EP-PF administration preserved the GVT effect (**Figures 10A–C**).

Beyond preserving GVT effects, we further investigated whether PF exerted direct anti-hematological tumor activity using multiple hematological malignancy cell lines and xenograft models. Results showed that PF significantly inhibited the proliferation of leukemia (HL-60, KG-1a, K-562) and lymphoma (SU-DHL-6) cell lines (**Supplementary Figures 5A–D**). Moreover, PF also promoted tumor cell apoptosis (**Supplementary Figures 5E–H**). Furthermore, subcutaneous tumor xenograft assay demonstrated that PF significantly inhibited the growth of KG-1a and SU-DHL-6 cells in nude mice (**Supplementary Figures 5I–J**). Meanwhile, PF also significantly upregulated the expression of CASP3 and CASP9, and downregulated the expression of BCL2 in xenograft tumors, thereby inducing apoptosis of tumor cells (**Supplementary Figure 5K**). These results suggested that PF exerted direct anti-hematological tumor effects.

## Discussion

To date, aGVHD remains the major complication of allo-HSCT, with severe immune responses and target organ damage, resulting in transplant failure and poor prognosis (5). The combination of CNIs and MTX is frequently used clinically through suppressing T cell activation. However, this regimen is usually accompanied by significant renal toxicity, heightened risks of life-threatening infections, and suboptimal efficacy in Haplo-HCT (59–61). On the other hand, how to preserve the GVT effect at the same time when preventing aGVHD is another critical challenge (62, 63). In this context, we conducted a screening to explore the potential natural products for aGVHD prevention, and PF subsequently emerged as a prominent candidate. We constructed aGVHD mouse models and performed a series of experiments to verify the preventive effects of PF in aGVHD. Our results demonstrated that PF could significantly alleviate aGVHD-associated symptoms and prolong survival, by reducing T cell infiltration, balancing cytokines and chemokines, relieving gut barrier damage, as well as restoring gut microbiota. Interestingly, PF improved the hematopoietic potential of graft HSPCs, and promoted the hematopoietic reconstitution in recipients after transplantation. Moreover, PF administration preserved the GVT activity against lymphoma cells in mouse model, suggesting that PF prophylaxis for aGVHD may not increase the risk of primary disease relapse.

PF is a natural monoterpene glycoside derived from the root of *Radix Paeoniae Alba* (Bai Shao) and *Radix Paeoniae Rubra* (Chi Shao), with characteristics of rapid absorption, broad tissue distribution, and low toxicity (64). Previous studies indicated that PF promoted hematopoietic recovery in irradiation-induced myelosuppression mice. For example, Zhu et al. demonstrated that PF significantly increased the white blood cell (WBC) counts, reversed the atrophy of thymus, and elevated the serum levels of hematopoietic stimulating factors such as interleukin-3 (IL-3), granulocyte colony-stimulating factor (G-CSF), and granulocyte-macrophage colony-stimulating factor (GM-CSF) in myelosuppression induced by chemotherapy or irradiation (27, 65). Liang et al. reported that PF increased colony counts of 4 types of hematopoietic progenitor cells, including colony-forming unit-granulocyte/macrophage (CFU-GM), colony-forming unit-erythroid (CFU-E), burst-forming unit-erythroid (BFU-E), and colony-forming unit-mixed (CFU-mix), and elevated peripheral leukocyte counts after irradiation (66). However, the specific mechanisms still remain unclear, and its hematopoietic-promoting effects in aGVHD also have not been validated. Herein, we found that PF significantly increased the routine blood cell counts, BMMC and HSPC numbers, as well as the expression of hematopoietic differentiation-associated transcription factors in aGVHD mice. Moreover, PF could also increase the HSPC numbers, and upregulated the transcription factors involved in the maintenance, self-renewal, and lineage differentiation in HSPCs. In addition, PF potentially activated multiple metabolic pathways in HSPCs, including glucose, fatty acid and amino acid metabolism, providing energy for their proliferation (47, 67). Given the benefits of PF on hematopoiesis, we proposed an entire-process PF management strategy involving PF administration to both donors and recipients, to investigate whether this approach could further enhance the efficacy of aGVHD prevention. In most subsequent experiments, EP-PF exhibited superior protective effects compared with PF administration for recipients only, highlighting the potential application value of this strategy.

A potential concern is whether the hematopoietic-promoting effects of PF may accelerate the production of donor-derived immune cells, thereby deteriorating aGVHD. However, our results did not support this suspicion, as PF, especially EP-PF, alleviated aGVHD-induced target organ damage, limited donor T cell infiltration, and decreased inflammatory cytokine levels while promoting hematopoietic reconstitution. These findings suggested that PF primarily restored graft hematopoietic potential rather than promoting the uncontrolled expansion of pathogenic alloreactive T cells. Clinical analyses also showed that the early recovery of absolute lymphocyte count (ALC), natural killer (NK) cells, or CD8^+^ T cells was associated with improved survival, reduced infection, and preserved GVT effects without necessarily increasing graft-versus-host disease (68–71). One possible explanation was that the immune cells newly generated from HSPCs underwent a tolerogenic maturation process before becoming functional effector cells. Previous studies demonstrated that, after allo-HSCT, thymic-dependent T cell development and central tolerance mechanisms, particularly negative selection, could promote the immune tolerance of newly generated donor-derived T cells toward alloantigens (72, 73). This possibility was also conceptually supported by the clinical experience of Haplo-HCT using the Beijing Protocol, in which immune tolerance could be promoted even under MHC-mismatched conditions through G-CSF- and anti-thymocyte globulin (ATG)-based immune modulation (74, 75).

Currently, the anti-inflammatory effects of PF have been reported in multiple immune diseases (24, 76, 77). In allergic contact dermatitis, PF markedly ameliorated T cell-mediated skin tissue damage by inhibiting T cell activation through suppressing NF-κB pathway activation and IFN-γ production (24). In autoimmune encephalomyelitis, PF attenuated spinal cord demyelination and inflammatory cell infiltration by reducing the secretion of cytokines IL-6 and interleukin-17 (IL-17), and suppressing T helper 17 (Th17) cell differentiation (76). In autoimmune myocarditis, PF was demonstrated to mitigate myocardial tissue necrosis and reduce serum levels of TNF-α, IL-6, IFN-γ and interleukin-21 (IL-21), by decreasing follicular helper T cell proportion through inhibiting C-X-C motif chemokine receptor 5 (CXCR5)/MAPK signaling (77). However, whether PF exerts similar anti-inflammatory effects in aGVHD remains unanswered. Herein, we provided experimental evidence that PF exerted potent anti-inflammatory effects in aGVHD. PF markedly reduced the accumulation of donor-derived CD4^+^ and CD8^+^ T cells in the gut, and partially restored the CD4^+^/CD8^+^ T cell balance. Transcriptomic analysis further showed that EP-PF reversed a large proportion of aGVHD-induced gene expression abnormalities in donor T cells, especially those associated with chemokine signaling, JAK-STAT signaling, TNF signaling, and cytokine-cytokine receptor interaction. Consistently, PF decreased serum levels of pro-inflammatory cytokines (TNF-α, IFN-γ, IL-2, IL-6, IL-17A), increased the levels of immunomodulatory cytokines (IL-4, IL-10), and suppressed the overproduction of multiple chemokines (CCL2, CCL3, CCL4, CCL5, CXCL1). However, our results showed that PF did not significantly alter the proportions of CD25^+^Foxp3^+^ Tregs and CD11b^+^Gr-1^+^ MDSCs, two immunosuppressive cell populations implicated in the control of alloreactive T cell responses and aGVHD development (78–82). Overall, these findings provided important preclinical evidence supporting PF as a potential prophylactic agent for aGVHD.

Gut damage occurs in over 50% of aGVHD patients and represents a serious threat to the patient life (83). Multiple studies have suggested that PF plays gut protective roles in different diseases (84, 85). In ulcerative colitis, PF promoted intestinal stem cell differentiation and epithelial regeneration by activating phosphoinositide 3-kinase/protein kinase B (PI3K/AKT) signaling pathway (84). Moreover, PF enhanced liver kinase B1/adenosine monophosphate-activated protein kinase (LKB1/AMPK) signaling-mediated gut autophagy, thereby mitigating morphological damage, oxidative stress, inflammatory response and apoptosis in gut ischemia-reperfusion injury (85). Furthermore, PF could alleviate mucous damage, inflammatory cell infiltration and gut edema, as well as recover blood supply by inhibiting apoptosis signal-regulating kinase 1 and tissue factor axis in radiation enteritis (86). Nevertheless, whether PF improves aGVHD-induced gut damage remains uninvestigated. In this study, we demonstrated that PF administration reduced diarrhea severity, as reflected by lower diarrhea rates, diarrhea index, and fecal moisture. Histological analysis also showed that PF improved villus disruption, crypt destruction, goblet cell loss, and inflammatory cell infiltration in the small intestine and colon, with the most pronounced effects observed in the EP-PF group. In addition, PF decreased intestinal permeability and restored the expression of tight junction proteins, including Claudin-1, Occludin, and ZO-1. PF also reversed the abnormal expression of apoptosis-related molecules, with reduced Casp3, Casp9, and Bax expression and increased Bcl2 expression in gut tissues. These results indicated that PF protected the gut from aGVHD-induced injury by maintaining epithelial barrier integrity and limiting intestinal apoptosis.

Dysbiosis of gut microbiota is a hot research topic at present which closely associated with gut damage, but its impacts on aGVHD remains understudied (55, 87). Herein, we found significant loss of gut microbial diversity and obvious structural dysbiosis in aGVHD mice, while these alterations could be significantly reversed by PF, indicating the protective roles of paeoniflorin in aGVHD-induced gut damage. Of note, we found that *Lactobacillus* was expanded in the aGVHD group and decreased after PF administration, while its expansion was significantly associated with the reduction of anti-inflammatory cytokine IL-10 according to a previous study (88). This result suggested that PF-mediated restoration of gut microbiota may drive the upregulation of anti-inflammatory cytokines, but needing further validation. Subsequent differential metabolic pathway analysis revealed that butirosin and neomycin biosynthesis pathway was upregulated, and bacterial invasion of epithelial cells pathway was downregulated. Notably, butirosin and neomycin are aminoglycoside antibiotics and primarily target gram-negative bacteria (e.g. Escherichia coli, Pseudomonas aeruginosa, Enterobacter aerogenes), whose expansion has been demonstrated to aggravate aGVHD-associated gut damage, suggesting paeoniflorin may promote the specific antibiotics biosynthesis to inhibit pathogenic bacteria growth (89–92). Taken together, these findings suggested that PF-mediated restoration of gut microbiota may contribute to its protective effects against aGVHD-induced gut damage.

The recurrence of primary tumor after transplantation poses another threat to the survival of patients (1). The GVT effect represents a key therapeutic benefit of allo-HSCT, as donor immune cells recognize and eliminate residual malignant cells after transplantation (93, 94). Therefore, an ideal strategy for aGVHD prevention should reduce harmful alloreactivity against normal tissues with preserving the GVT effect (95). For example, Su et al. showed that high-dose icariin administered early after allo-HSCT significantly alleviated murine aGVHD, while preserving donor cell-mediated GVT effects against both acute myeloid leukemia and T-cell acute lymphoblastic leukemia (35). In addition, Xu et al. reported that cysteine-rich secretory protein LCCL domain containing 2 (CRISPLD2)-overexpressing human umbilical cord-derived mesenchymal stem cells reduced aGVHD severity in an A20 tumor-bearing allo-HSCT model, and the treated mice showed only transient tumor growth, successful tumor clearance, and no obvious tumor relapse (38). Similarly, in our GVT model, EP-PF did not weaken the donor cell-mediated tumor control and survival benefit, as reflected by comparable survival outcomes and peripheral blood GFP^+^ A20 cell proportions between the GVT and GVT+EP-PF groups. These results suggested that PF could alleviate aGVHD while preserving donor cell-mediated GVT activity. Not only that, we also found that PF exerted direct anti-hematological tumor effects by inhibiting cell proliferation and inducing cell apoptosis *in vitro* and *in vivo*.

Several challenges should be addressed before the clinical translation of PF. Primarily, our findings were mainly based on murine aGVHD models, and the protective effects of PF need further validation in larger preclinical models and clinical trials. Meanwhile, the optimal dose, administration route, and administration window of PF after allo-HSCT remain to be determined. Moreover, although PF showed no obvious toxicity in our study, its long-term safety and potential interactions with standard aGVHD prophylactic drugs require further evaluation. The mechanisms by which PF regulates donor T cell responses, hematopoietic recovery, and gut microbiota also need deeper investigation. Finally, whether PF can preserve GVT activity across different tumor types and transplantation settings should be confirmed. Addressing these issues will be important for promoting PF from experimental research to clinical application.

In conclusion, we highlight the preventive effects of PF on aGVHD through multiple pathways and the preservation of GVT effects. Entire-process paeoniflorin management regimen can further improve hematopoietic potential, alleviate aGVHD severity and prolong post-transplant survival. Paeoniflorin, the herb-derived natural single compound, has shown great potential in clinical application of allo-HSCT.

## Data Availability

The original contributions presented in the study are included in the article/**Supplementary Material**. Further inquiries can be directed to the corresponding authors.

## Glossary

7-AAD: 7-Aminoactinomycin D
aGVHD: Acute graft-versus-host disease
ALC: Absolute lymphocyte count
allo-HSCT: Allogeneic hematopoietic stem cell transplantation
ARRIVE: Animal Research Reporting of *In Vivo* Experiments
ASVs: Amplicon sequence variants
ATCC: American Type Culture Collection
ATG: Anti-thymocyte globulin
ATP: Adenosine triphosphate
B6: C57BL/6
BCA: Bicinchoninic acid
BFU-E: Burst-forming unit-erythroid
BMCs: Bone marrow cells
BMMC/BMMCs: Bone marrow mononuclear cell(s)
BMT: Bone marrow transplantation
BP: Biological process
CC: Cellular component
CCK-8: Cell Counting Kit-8
CCL2: C-C motif chemokine ligand 2
CCL3: C-C motif chemokine ligand 3
CCL4: C-C motif chemokine ligand 4
CCL5: C-C motif chemokine ligand 5
CD11b: Cluster of differentiation 11b
CD25: Cluster of differentiation 25
CD3: Cluster of differentiation 3
CD4: Cluster of differentiation 4
CD8: Cluster of differentiation 8
CDC42/JNK: Cell division cycle 42/c-Jun N-terminal kinase
cDNA: Complementary DNA
CFU-E: Colony-forming unit-erythroid
CFU-GM: Colony-forming unit-granulocyte/macrophage
CFU-mix: Colony-forming unit-mixed
c-Kit: KIT proto-oncogene receptor tyrosine kinase
CNI/CNIs: Calcineurin inhibitor(s)
CO_2_: Carbon dioxide
CRISPLD2: Cysteine-rich secretory protein LCCL domain containing 2
CsA: Cyclosporin A
CTAB: Cetyltrimethylammonium bromide
CXCL1: C-X-C motif chemokine ligand 1
CXCR5: C-X-C motif chemokine receptor 5
DAVID: Database for Annotation, Visualization and Integrated Discovery
DEGs: Differentially expressed genes
DMPNN: Directed message passing neural network
DNase I: Deoxyribonuclease I
ECL: Enhanced chemiluminescence
EDTA: Ethylenediaminetetraacetic acid
EP-PF: Entire-process paeoniflorin
FC: Fold change
FITC-Dextran: Fluorescein isothiocyanate-dextran
FoxO: Forkhead box O
Foxp3: Forkhead box P3
G-CSF: Granulocyte colony-stimulating factor
GFP: Green fluorescent protein
GM-CSF: Granulocyte-macrophage colony-stimulating factor
GO: Gene Ontology
Gr-1: Granulocyte differentiation antigen-1
GSEA: Gene set enrichment analysis
GVT: Graft-versus-tumor
H&E: Hematoxylin and eosin
Hacc: Hydrogen-bond acceptors
Haplo-HCT: Haploidentical hematopoietic cell transplantation
HBSS: Hanks’
Hdon: Hydrogen-bond donors
HIF-1: Hypoxia-inducible factor 1
HRP: Horseradish peroxidase
HSPC/HSPCs: Hematopoietic stem/progenitor cell(s)
IFN-γ: Interferon-gamma
IL-10: Interleukin-10
IL-17: Interleukin-17
IL-17A: Interleukin-17A
IL-2: Interleukin-2
IL-21: Interleukin-21
IL-3: Interleukin-3
IL-4: Interleukin-4
IL-6: Interleukin-6
JAK-STAT: Janus kinase-signal transducer and activator of transcription
KEGG: Kyoto Encyclopedia of Genes and Genomes
LEfSe: Linear discriminant analysis effect size
Lin: Lineage
LKB1/AMPK: Liver kinase B1/adenosine monophosphate-activated protein kinase
logP: Logarithm of the octanol-water partition coefficient
LSK: Lin^-^Sca-1^+^c-Kit^+^
MAPK: Mitogen-activated protein kinase
MDSC/MDSCs: Myeloid-derived suppressor cell(s)
MF: Molecular function
MHC: Major histocompatibility complex
mRNA: Messenger RNA
MTX: Methotrexate
MW: Molecular weight
NES: Normalized enrichment score
NEUT: Neutrophil
NF-κB: Nuclear factor kappa-B
NK: Natural killer
ns: Not significant
PBS: Phosphate-buffered saline
PF: Paeoniflorin
PFA: Paraformaldehyde
PF-HD: Paeoniflorin high-dose group
PF-LD: Paeoniflorin low-dose group
PF-MD: Paeoniflorin medium-dose group
PI3K/AKT: Phosphoinositide 3-kinase/protein kinase B
PLT: Platelet
PT-Cy: Post-transplantation cyclophosphamide
PVDF: Polyvinylidene difluoride
QIIME 2: Quantitative Insights Into Microbial Ecology 2
qPCR: Quantitative polymerase chain reaction
RBC: Red blood cell
RIN: RNA integrity number
RIPA: Radioimmunoprecipitation assay
RNA-Seq: RNA sequencing
Sca-1: Stem cell antigen-1
SDS-PAGE: Sodium dodecyl sulfate-polyacrylamide gel electrophoresis
SPCs: Splenic cells
SPF: Specific pathogen-free
TBST: Tris-buffered saline with Tween-20
Th17: T helper 17
Th2: T helper 2
TNF-α: Tumor necrosis factor-alpha
TPSA: Topological polar surface area
Treg/Tregs: Regulatory T cell(s)
WBC: White blood cell
ZO-1: Zonula occludens-1.
