# Supplementary material for: Paeoniflorin prevents acute graft-versus-host disease while preserving graft-versus-tumor effects
Source: Front Immunol. 2026 Sep 8;17:1937379. doi: 10.3389/fimmu.2026.1937379 (PMC13593478; doi:10.3389/fimmu.2026.1937379)
Supplement: Supplementary file 1 [file SupplementaryFile1.docx]

**Supplement Table 1. Criteria of clinical score assessment in aGVHD mice**

| **Manifestations** | **Score 0** | **Score 1** | **Score 2** |
| --- | --- | --- | --- |
| Body weight loss | <10% | 10%~25% | >25% |
| Posture | Normal | Hunching noted only at rest | Severe hunching impaired movement |
| Activity | Normal | Mild to moderately decreased | Stationary unless stimulated |
| Fur texture | Normal | Mild to moderate ruffling | Severe ruffling/poor grooming |
| Skin integrity | Normal | Scaling of paws/tail | Obvious areas of denuded skin |

**Supplement Table 2. Criteria of pathological score assessment in aGVHD mice**

| **Tissue type** | | **0** | **1** | **2** | **3** | **4** |
| --- | --- | --- | --- | --- | --- | --- |
| Skin | Epidermis | Normal | Focal interface damage involving <20% of the section, with occasional necrotic keratinocytes | Widespread interface damage involving >20% of the section | —— | —— |
|  | Dermis | Normal | Slight alteration with a mild increase in collagen density | Marked increase in collagen density | —— | —— |
|  | Inflammation | Normal | Focal inflammatory infiltrates | Widespread inflammatory infiltrates | —— | —— |
|  | Subcutaneous fat | Normal | Reduced number of normal adipocytes | Serous fat atrophy | —— | —— |
|  | Hair follicles | Normal | 1-5 follicles per linear millimeter | <1 follicle per linear millimeter | —— | —— |
| Gut | Crypt apoptosis | Rare or absent apoptotic bodies | Occasional apoptotic bodies per 10 crypts | A few apoptotic bodies per 10 crypts | The majority of crypts contain one apoptotic body | The majority of crypts contain >1 apoptotic body |
|  | Inflammation | Normal | Mild | Moderate | Severe, without ulceration | Severe, with ulceration |
| Liver | Bile duct injury and inflammation | Normal | A few portal tracts involved, with mild changes | Numerous portal tracts involved, but with only mild disease | Injury involving the majority of portal tracts | Severe involvement of most portal tracts |
| Lung | Periluminal infiltrates (around airways/vessels) | Normal | 1-3 cell diameters thick | 4-10 cell diameters thick | >10 cell diameters thick | —— |
|  | Pneumonitis (alveolar/interstitial) | Normal | Increased cells, only visible at high magnification (×400) | Easily seen cellular infiltrate or interstitial thickening | Consolidation by inflammatory cells and interstitial thickening | —— |

**Supplement Table 3. Primer sequences for qPCR**

| **Genes** | **Species** | **Direction** | **Sequence** |
| --- | --- | --- | --- |
| Gata1 | Murine | Forward | TGTCCTCACCATCAGATTCCA |
|  |  | Reverse | TCCCTCCATACTGTTGAGCAG |
| Klf1 | Murine | Forward | GATCGCCGGAGACGCAGGCT |
|  |  | Reverse | TCCCCAGTCCTTGTGCAGGA |
| Cebpa | Murine | Forward | GCGGGAACGCAACAACATC |
|  |  | Reverse | GTCACTGGTCAACTCCAGCAC |
| Runx1 | Murine | Forward | TGGTGGAGGTACTAGCTGACC |
|  |  | Reverse | CGAGTAGTTTTCATCGTTGCCTG |
| Tal1 | Murine | Forward | ACTAGGCAGTGGGTTCTTTGG |
|  |  | Reverse | CCCGGCTGTTGGTGAAGAT |
| Erg | Murine | Forward | CCAGCAGCTCATATTAAGGAGG |
|  |  | Reverse | CGTTCCGTAGGCACACTCA |
| Casp3 | Murine | Forward | CTGACTGGAAAGCCGAAACTC |
|  |  | Reverse | CGACCCGTCCTTTGAATTTCT |
| Casp9 | Murine | Forward | TCAGGGGACATGCAGATATGG |
|  |  | Reverse | TTGGCAGTCAGGTCGTTCTTC |
| Bcl2 | Murine | Forward | GCTACCGTCGTGACTTCGC |
|  |  | Reverse | CCCCACCGAACTCAAAGAAGG |
| Bax | Murine | Forward | AGACAGGGGCCTTTTTGCTAC |
|  |  | Reverse | AATTCGCCGGAGACACTCG |
| Actb | Murine | Forward | GTGACGTTGACATCCGTAAAGA |
|  |  | Reverse | GCCGGACTCATCGTACTCC |
| CASP3 | Human | Forward | CATGGAAGCGAATCAATGGACT |
|  |  | Reverse | CTGTACCAGACCGAGATGTCA |
| CASP9 | Human | Forward | CTTCGTTTCTGCGAACTAACAGG |
|  |  | Reverse | GCACCACTGGGGTAAGGTTT |
| BCL2 | Human | Forward | ACTGGCTCTGTCTGAGTAAG |
|  |  | Reverse | CCTGATGCTCTGGGTAAC |
| ACTB | Human | Forward | CATGTACGTTGCTATCCAGGC |
|  |  | Reverse | CTCCTTAATGTCACGCACGAT |

**Supplement Table 4. Impacts of PF on hepatotoxicity and nephrotoxicity in B6-to-BALB/c transplantation.**

| **Index** | **BMT (n=6)** | **BMT+PF-MD (n=6)** | **p value** |
| --- | --- | --- | --- |
| **Renal function** |  |  |  |
| UREA (mmol/L) | 6.700±1.170 | 6.483±0.850 | 0.7212 |
| CREA (μmol/L) | 14.67±2.338 | 16.33±2.160 | 0.2286 |
| UA (μmol/L) | 156.3±11.55 | 162.2±18.93 | 0.5338 |
| **Liver function** |  |  |  |
| ALT (IU/L) | 37.50±6.285 | 36.83±4.708 | 0.8394 |
| AST (IU/L) | 91.33±8.335 | 89.50±10.63 | 0.7465 |
| ALP (U/L) | 215.2±12.84 | 227.5±20.53 | 0.2406 |
| TP (g/L) | 48.47±1.350 | 49.23±1.229 | 0.3279 |
| ALB (g/L) | 20.55±1.487 | 20.90±1.644 | 0.7071 |
| GLB (g/L) | 28.15±1.050 | 28.32±1.068 | 0.7908 |

**Abbreviation**: UREA: urea; CREA: creatinine; UA: uric acid; ALT: alanine aminotransferase; AST: aspartate aminotransferase; ALP: alkaline phosphatase; TP: total protein; ALB: albumin; GLB: globulin.

**Supplement Table 5. GSEA analysis of feature genes related to glucose metabolism, fatty acid metabolism, and amino acid metabolism in HSPCs**

| **Gene Set** | **NES** | **p value** | **Leading Edge** | **Enriched Core Genes** |
| --- | --- | --- | --- | --- |
| Glycolysis / Gluconeogenesis (mmu00010) | 1.6192 | 0.0061 | Tags=47%, List=21%, Signal=37% | Aldob, Eno4, Ldhc, Adh1, Aldh3b1, Hk3, Pkm, Ldhb, Gpi1, Eno1b, Adpgk, Ldhal6b, Eno1, Pgam1, Aldoart1, Aldoa, Aldh3b3, Pgm2, Aldh2, Galm, G6pc3, Aldoart2, Pfkp, Ldha, Fbp1, Pgk1, Tpi1 |
| Pentose phosphate pathway (mmu00030) | 1.8409 | 0.0003 | Tags=56%, List=20%, Signal=45% | Aldob, Rgn, Prps1l1, Pgd, G6pdx, Gpi1, H6pd, Tktl1, Aldoart1, Aldoa, G6pd2, Tkt, Pgm2, Taldo1, Aldoart2, Rpe, Pfkp, Fbp1 |
| Arachidonic acid metabolism (mmu00590) | 1.7710 | 0.0008 | Tags=42%, List=12%, Signal=37% | Cyp2b10, Cyp2e1, Cyp2c23, Plb1, Pla2g10, Alox12, Ptgis, Ptges, Lta4h, Cyp4a31, Ptgs1, Alox5, Alox15, Ptgr1, Pla2g2d, Pla2g4e, Pla2g4d, Pla2g2f, Pla2g2e, Pla2g12b, Cyp2c29, Cyp2b9 |
| Alpha-linolenic acid metabolism (mmu00592) | 1.5080 | 0.0304 | Tags=38%, List=10%, Signal=34% | Plb1, Pla2g10, Pla2g2d, Pla2g4e, Pla2g4d, Pla2g2f, Pla2g2e, Pla2g12b |
| Linoleic acid metabolism (mmu00591) | 1.7487 | 0.0018 | Tags=52%, List=12%, Signal=46% | Cyp2e1, Cyp2c23, Cyp3a44, Plb1, Pla2g10, Alox15, Pla2g2d, Pla2g4e, Pla2g4d, Pla2g2f, Pla2g2e, Pla2g12b, Cyp2c29 |
| Biosynthesis of amino acids (mmu01230) | 1.5153 | 0.0095 | Tags=34%, List=21%, Signal=27% | Aldob, Prps1l1, Eno4, Cbs, Pkm, Cth, Eno1b, Eno1, Tktl1, Pgam1, Aldoart1, Aldoa, Bcat2, Tkt, Taldo1, Idh1, Got1, Aldoart2, Rpe, Pfkp, Pgk1, Tpi1, Asns |
| Tyrosine metabolism (mmu00350) | 1.5068 | 0.0327 | Tags=24%, List=8%, Signal=22% | Aox1, Aoc3, Tyr, Fah, Adh1, 4930438A08Rik, Aldh3b1 |

**Abbreviation**: NES: normalized enrichment score.

**Supplement Figure 1. PF bound to multiple aGVHD-related signaling molecules based on molecular docking simulations.** (A-G) Molecular docking simulation between paeoniflorin and (A) JAK1, (B) JAK2, (C) JAK3, (D) STAT1, (E) STAT3, (F) IL6, (G) NFKB1. The red molecule represented PF. The yellow molecule represented specific protein. The purple structure represented the interactive amino acid residues. The binding energy < -5.0 kcal/mol was considered as stable binding.

**Supplement Figure 2. PF alleviated aGVHD and enhanced hematopoietic reconstitution in mouse model (extended).** (A) The evaluation of CD3^+^ T cell proportion in splenic cells of B6 or BALB/c mice. (B) The schematic presentation of BALB/c-to-B6 aGVHD mouse model. (C) The proportion of donor-derived H-2Kd positive cells in bone marrow was evaluated by flow cytometry at day 40 or upon mortality (n = 10 in each group). (D-F) The clinical scores (D), survival status (E), and body weight (F) were evaluated from day 0 to day 40 (n = 10 in each group). Data from day 0 to day 6 were excluded for statistical analysis. Intergroup comparison of clinical scores was performed using the Kruskal-Wallis test followed by Dunn’s post tests. (G) The flow cytometric gating strategy for LSK cells in bone marrow. * P < 0.05 (vs. BMT group), ** P < 0.01 (vs. BMT group), # P < 0.05 (vs. aGVHD group), ## P < 0.01 (vs. aGVHD group).

**Supplement Figure 3. PF reduced donor T cell infiltration in gut (extended).** (A) The flow cytometric gating strategy for donor T cells in gut. (B-C) The numbers of (B) CD3^+^CD4^+^ T cells and (C) CD3^+^CD8^+^ T cells in each group (n = 5). (D-E) The MDSC proportions (D) and numbers (E) in each group (n = 5). (F) The intestinal representative flow cytometry plots of CD11b^+^Gr-1^+^ MDSCs. * P < 0.05 (vs. BMT group), ** P < 0.01 (vs. BMT group), # P < 0.05 (vs. aGVHD group), ## P < 0.01 (vs. aGVHD group).

**Supplement Figure 4. PF rescued gene expression dysregulation in splenic donor T cells (extended).** (A) KEGG enrichment of DEGs in donor CD8^+^ T cells in aGVHD vs. BMT comparison (left) and EP-PF vs. aGVHD comparison (right). (B) KEGG enrichment of DEGs in donor CD4^+^ T cells in aGVHD vs. BMT comparison (left) and EP-PF vs. aGVHD comparison (right).

**Supplement Figure 5. PF exerted direct anti-hematological tumor activity.** (A-D) PF inhibited the proliferation of (A) HL-60, (B) KG-1a, (C) K-562, and (D) SU-DHL-6 cells (n = 5). (E-H) PF promoted cell apoptosis in (E) HL-60, (F) KG-1a, (G) K-562, and (H) SU-DHL-6 cells (n = 3). (I-J) PF inhibited (I) KG-1a and (J) SU-DHL-6 tumor growth in nude mice (n = 6). (K) PF promoted the expression of CASP3 and CASP9, and downregulated the expression of BCL2 in KG-1a and SU-DHL-6 xenograft tumor cells (n = 6). * P < 0.05, ** P < 0.01.
